# Assessing the influence of health systems on Type 2 Diabetes Mellitus awareness, treatment, adherence, and control: A systematic review

**DOI:** 10.1371/journal.pone.0195086

**Published:** 2018-03-29

**Authors:** Suan Ee Ong, Joel Jun Kai Koh, Sue-Anne Ee Shiow Toh, Kee Seng Chia, Dina Balabanova, Martin McKee, Pablo Perel, Helena Legido-Quigley

**Affiliations:** 1 Saw Swee Hock School of Public Health, National University of Singapore, Singapore, Singapore; 2 Yong Loo Lin School of Medicine, National University of Singapore, Singapore, Singapore; 3 Division of Endocrinology, Department of Medicine, National University Health System, Singapore, Singapore; 4 London School of Hygiene and Tropical Medicine, London, United Kingdom; 5 World Heart Federation, Geneva, Switzerland; Universidade de Mogi das Cruzes, BRAZIL

## Abstract

**Background:**

Type 2 Diabetes Mellitus (T2DM) is reported to affect one in 11 adults worldwide, with over 80% of T2DM patients residing in low-to-middle-income countries. Health systems play an integral role in responding to this increasing global prevalence, and are key to ensuring effective diabetes management. We conducted a systematic review to examine the health system-level factors influencing T2DM awareness, treatment, adherence, and control.

**Methods and findings:**

A protocol for this study was published on the PROSPERO international prospective register of systematic reviews (PROSPERO 2016: CRD42016048185). Studies included in this review reported the effects of health systems factors, interventions, policies, or programmes on T2DM control, awareness, treatment, and adherence. The following databases were searched on 22 February 2017: Medline, Embase, Global health, LILACS, Africa-Wide, IMSEAR, IMEMR, and WPRIM. There were no restrictions on date, language, or study designs. Two reviewers independently screened studies for eligibility, extracted the data, and screened for risk of bias. Thereafter, we performed a narrative synthesis. A meta-analysis was not conducted due to methodological heterogeneity across different aspects of included studies. 93 studies were included for qualitative synthesis; 7 were conducted in LMICs. Through this review, we found two key health system barriers to effective T2DM care and management: financial constraints faced by the patient and limited access to health services and medication. We also found three health system factors that facilitate effective T2DM care and management: the use of innovative care models, increased pharmacist involvement in care delivery, and education programmes led by healthcare professionals.

**Conclusions:**

This review points to the importance of reducing, or possibly eliminating, out-of-pocket costs for diabetes medication and self-monitoring supplies. It also points to the potential of adopting more innovative and integrated models of care, and the value of task-sharing of care with pharmacists. More studies which identify the effect of health system arrangements on various outcomes, particularly awareness, are needed.

## Introduction

The 2015 International Diabetes Federation’s Diabetes Atlas [1] reported that 415 million people worldwide, or one in 11 adults, has diabetes, with most having Type 2 Diabetes Mellitus (T2DM) [1]. Although the incidence and prevalence of T2DM varies by geographical region, with over 80% of T2DM patients residing in low-to-middle-income countries, T2DM prevalence has increased worldwide since 1980 [2]. Health systems play a crucial role in the response to this rising burden, preventing premature death and disability and improving quality of life [2, 3]. Yet, while the management of diabetes has been the subject of many systematic reviews [4–12], these have focused on particular interventions, models of care, or information technology support systems. To our knowledge, no systematic review assembles the evidence appraising the impact of health systems on management of type 2 diabetes mellitus (T2DM). To address this gap, we systematically review the literature examining the health system-level factors influencing T2DM awareness, treatment, adherence, and control, and make recommendations for future research and policy considerations.

## Methods

A protocol for this study was published on the PROSPERO international prospective register of systematic reviews (PROSPERO 2016: CRD42016048185). There are several ways in which the findings could be arranged but, as we were taking a health systems perspective, we used a conceptual framework developed by Balabanova and colleagues [13], which has been used to understand aspects of systems that hinder the effective management of non-communicable diseases [13, 14]. This framework identifies physical resources (e.g. healthcare facilities, pharmaceuticals, technologies), human resources (e.g. trained health workers), intellectual resources (e.g. clinical practice guidelines), and social resources (e.g. social capital, organisational measures to enhance collaboration) as necessary elements of a health system response to chronic disease challenges. The framework addresses inputs that underpin health system functioning in three key areas, namely service delivery, healthcare financing, and governance. These areas are recognised as critical elements of effective health system functioning by the World Health Organization [15]. In this review, following Gilson and colleagues, we define governance as: everyday actions and decisions that translate policy intentions into practice, which are “filtered through relationships, underpinned by values and norms, influenced by organisational structures and resources, and embedded in historical and socio-political contexts” [16] that reinforce or challenge institutional exclusion and power inequalities.

**Fig 1:**
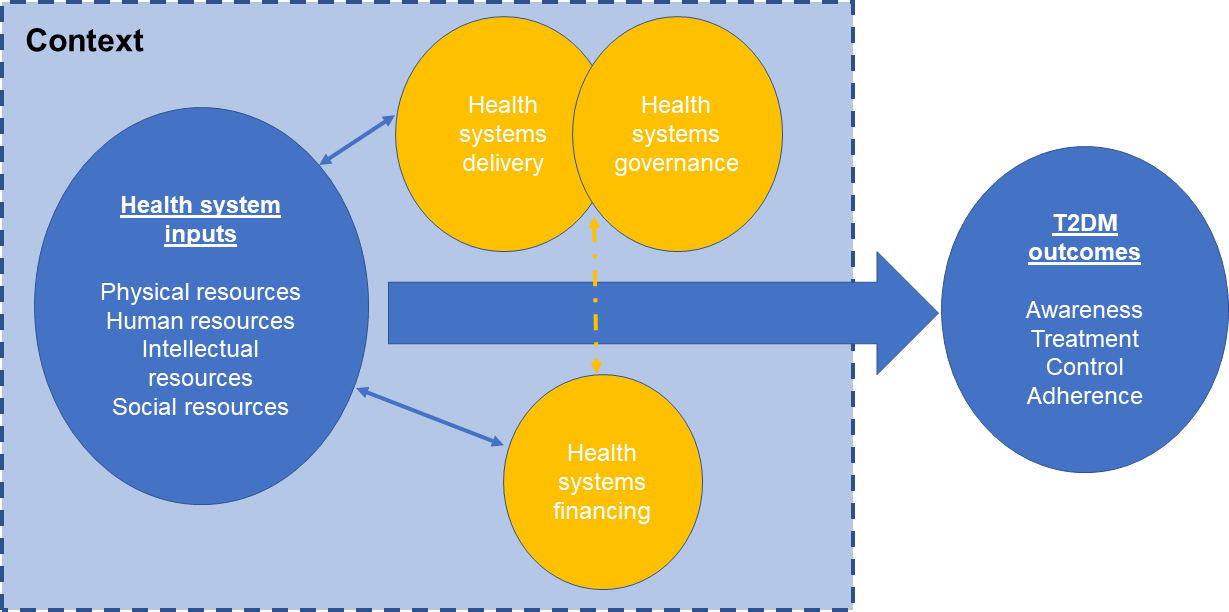
Conceptual framework.

Additionally, this framework takes account of the critical role of context in influencing the health system. In this manuscript, “context” refers to the socio-demographic, economic, and cultural setting in which health systems are embedded and operate. This framework is guided by the understanding that health and healthcare systems are complex adaptive systems that are dynamic, evolving, have multiple constituent parts, and are often unpredictable, exhibiting path dependency and feedback loops [17, 18]. As such, the ability of a health system to produce good outcomes does not rest on the robustness of disparate constituent “blocks”, but on the integration and alignment of inputs and system functioning components [19]. This approach has several advantages. First, it ensures that all of the elements of the health system are considered explicitly. Second, by taking a health systems approach rather than, for example, a clinical approach based on detection, treatment and control, it is designed to facilitate identification of actionable points by health policymakers. Third, it identifies important gaps in the evidence that will be needed to develop a comprehensive health system response to diabetes, thereby contributing to prioritisation of research efforts. The corresponding disadvantage is that there will be some areas where there is little or no research. The conceptual framework is shown in Fig 1 below.

### Inclusion and exclusion criteria

We included studies reporting the effects of macro and meso-level health system factors, interventions, policies, or programmes on T2DM control, awareness, treatment, and adherence. Box 1 outlines the definitions used and Box 2 details characteristics of included studies.

**Box 1. Definitions of included T2DM outcomes**

*T2DM awareness*: persons with clinically measured T2DM who have been diagnosed by a healthcare professional and are aware of their T2DM status

*T2DM treatment*: the use of at least one anti-diabetic medication in an individual with known T2DM

*Anti-diabetic medication adherence*: consistently taking antidiabetic medication as per regiment prescribed by a healthcare provider/professional

*T2DM control*: defined as the achievement of glycaemic control, blood pressure, and/or lipid control targets in individuals being managed for T2DM

**Box 2. Characteristics of included studies**

We included studies looking at any adult population, including general populations, populations receiving treatment, and populations of T2DM patients with related comorbidities, including hypertension and hyperlipidaemia. Included studies fell into two categories:

*Studies undertaken at the macro-level of the health system*: this includes, but is not confined to, national and international health policies, national healthcare financing structures, and national healthcare and health services delivery structures.

*Studies undertaken at the meso-level of the health system*: this includes, but is not restricted to, regional-level health systems/authorities, healthcare institutions (e.g. tertiary hospitals), and care organisations/networks (e.g. networks of primary care clinics)

Quantitative studies were included if they reported a measure of1 association between a health system element and at least one T2DM outcome. No date or language restrictions were applied. Translators were engaged to assist in determining the eligibility of non-English language literature. Translators helped with translation of titles, abstracts, and studies’ key findings. Studies evaluating interventions or programmes enacted at the micro-level (e.g. individual- or patient-level), such as those on the genetic profile of T2DM patients, were not included.

### Search strategy

The search strategy drew on that used by Maimaris and colleagues [20] in their health systems and hypertension systematic review. Key words (MeSH terms) and free-text terms were identified for each domain of our health systems framework and combined with search terms for T2DM outcomes to generate search strategies for Medline, Embase, and Global Health. In addition, modified searches were performed on Latin American and Carribean Health Siences Literature (LILACS), Africa-Wide, Index Medicus for the South-east Asian Region (IMSEAR), Index Medicus for the Eastern Mediterranean Region (IMEMR), and Western Pacific Rim Region Index Medicus (WPRIM). All databases were searched from inception to 22 February 2017.

### Study selection

Two reviewers independently screened search results by title and abstract for potential eligibility. Full-texts of potentially suitable articles were obtained and further screened by two reviewers. Disagreements were resolved by a third reviewer.

### Data extraction for study setting, methodology, and findings

A data extraction form was created in Microsoft Excel. Two reviewers independently extracted data on design, setting/context, health system domain/s investigated, outcomes and relevant findings, and checked for disparities.

### Risk of bias assessment

Two reviewers independently assessed included studies for risk of bias as low, medium, or high. For observational study designs (e.g. cross-sectional, case-control, cohort, pre-post, record/chart reviews) three domains were examined, as per Maimaris and colleagues in their systematic review [20]: selection bias, information bias (differential and non-differential misclassification), and confounding. Assessment of non-differential misclassification considered the reliability of the measure used to report T2DM outcomes. Studies assessed as having “low” or “high” risk of bias in most domains were classified as having low or high overall risk of bias respectively. Those where risk of bias was unclear in two domains, were classified as medium overall risk and those assessed to have unclear risk in three domains were classified as high overall risk.

The Cochrane risk of bias tool was used to assess randomised controlled trials, cluster randomised trials, and non-RCT, non-observational studies (e.g. trials that are not randomised or do not have a control group). Studies assessed as having low risk of bias across most domains were classified as low overall risk of bias. If risk was unclear in two to three domains and most domains were not classified as “high” or “low” risk of bias, the study was classified as medium overall risk of bias. Studies assessed to have unclear risk of bias in four domains were classified as having high overall risk of bias.

For quality assessment of qualitative studies, we used an adapted version of a checklist previously used in a series of mixed-methods systematic reviews [21, 22], comprising ten core criteria. Studies with a score of eight to ten were classified as having an overall low risk of bias, four to seven as overall medium risk of bias, and zero to three as overall high risk of bias.

### Assessment of context and complexity considerations

We assessed the extent to which included studies consider context and complexity, in respect of sociodemographic, political, economic, and/or cultural issues, as well as dynamic relationships between different health systems domains. We also explored how health systems interacted with contextual factors.

### Data analysis and synthesis

A narrative synthesis was performed. We categorised studies by health system domain and study setting, recognising that some investigated multiple domains simultaneously. Randomised controlled trials (RCTs) were considered the strongest design to establish causality, followed by cohort and case-control studies. Cross-sectional and ecological studies were not considered adequate to establish causality. We did not conduct a meta-analysis due to heterogeneity across study designs, populations, comparisons, analytical strategies, and outcomes [20].

## Results

**Fig 2:**
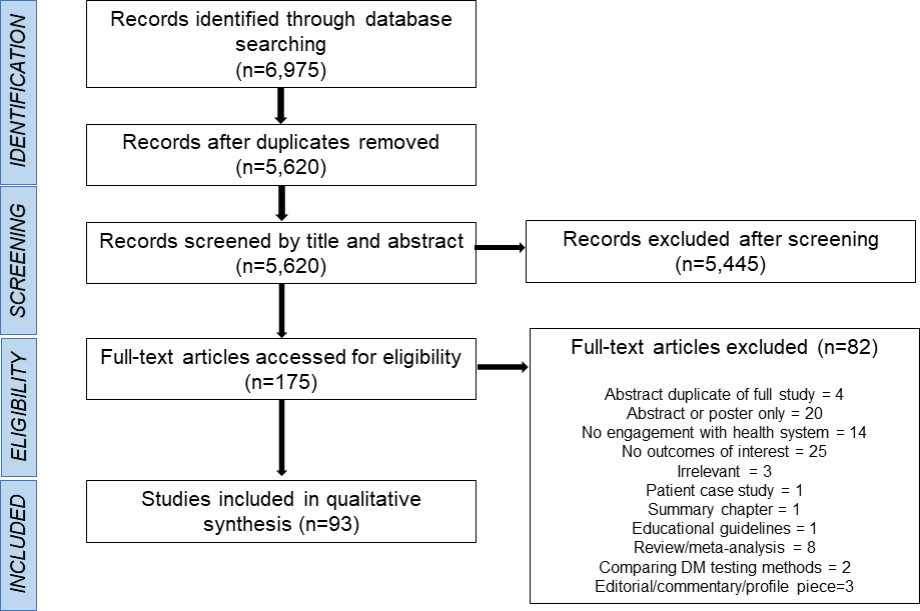
Adapted PRISMA flowchart.

We describe the screening process using an adapted Preferred Reporting Items for Systematic Reviews and Meta-Analyses (PRISMA) flowchart [23], shown in Fig 2 below.

Database searching identified 6,975 records, with 5,620 remaining after duplicate removal. After screening of titles and abstracts, 175 full-text articles were retrieved. 93 were included in the final qualitative synthesis. Of these 84 were quantitative. Of these, 21 were randomised controlled trials; one was a cluster randomised controlled trial; three were cluster randomised pragmatic trials; three were trials (i.e. trial designs with no mention of randomisation); 15 were cohort studies; one was a case-control study; 19 were cross-sectional studies; 14 were pre-post studies; six were record/chart reviews; and one was a time-series analysis. Of the remaining nine studies, six were qualitative and three used mixed methods. 77 (83%) of included studies were carried out in World Bank-classified high-income countries, nine in upper-middle income countries, and seven in lower-middle income countries.

### Geographical distribution of included studies

**Fig 3:**
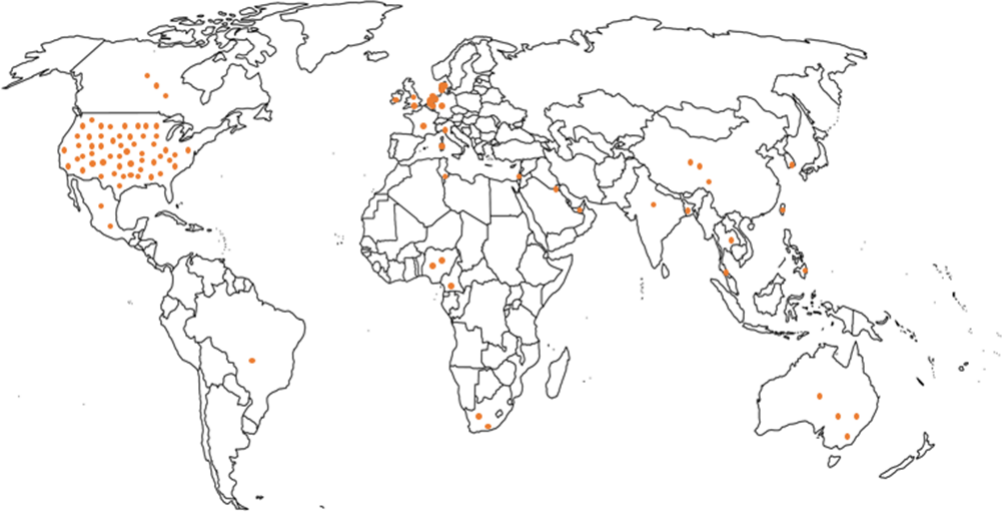
Geographical distribution of included studies.

As shown in Fig 3, most studies took place in North America (n = 53) and Europe (n = 16), with fewer in Asia (n = 10), Africa (n = 6), South America (n = 1), the Middle East (n = 3), and Australia (n = 4). Notably, all studies of healthcare financing were conducted in North America (n = 14).

### Risk of bias assessment

We conducted risk of bias assessment for all 93 articles. 28 studies had their risk of bias assessed using the Cochrane risk of bias tool. Of these, 22 were randomised controlled trials. 12 had high risk of bias [24–35], eight had medium risk of bias [36–43] and one had low risk of bias [44]. One cluster randomised controlled trial was assessed to have low risk of bias [45]. Three were cluster randomised pragmatic trials; two had high risk of bias [46, 47] and one had low risk of bias [48]. Three studies were trials: two had high risk of bias [49, 50] and one had medium risk of bias [51].

Among the 56 studies assessed as observational study designs, 19 were cross-sectional, of which seven had high risk of bias [52–58], four had a moderate risk of bias [59–62] and eight had low risk of bias [63–70]. 15 were cohort studies; one study had a high risk of bias [71], five had a medium risk of bias [72–77], and eight had low risk of bias [78–85]. One study used a case-control study design and had a moderate risk of bias [86]. 14 were pre-post studies; one had a high risk of bias [87], 11 had medium risk of bias [88–98], and two had low risk of bias [99, 100]. Six studies were record/chart reviews: two were assessed to have low risk of bias [101, 102] and four had medium risk of bias [103–106]. The one time-series study in this review had a medium risk of bias [107].

Six qualitative studies were assessed for risk of bias using a tool adapted from the Consolidated Criteria for Reporting Qualitative Studies (COREQ) checklist. One had a moderate risk of bias [108], and five had low risk of bias [109–113].

Risk of bias in the three mixed-methods studies was assessed separately for the quantitative and qualitative components. All three studies had quantitative components with a high risk of bias. One study had a moderate risk of bias for the qualitative component [114] and two had a low risk of bias for the qualitative component [115, 116].

### Context

32 of 93 included studies gave no detailed information about the socio-demographic, political, or economic context in which the study was conducted [26, 28–30, 34, 36, 37, 39, 40, 44, 45, 47, 52, 53, 55, 57, 64, 65, 68, 70, 72, 73, 84, 87, 89, 90, 95, 97, 101, 103, 105, 110]. 61 included studies that provided contextual information on various levels.

Three studies described the regional context, such as Sub-Saharan Africa and South America [49, 83, 99]. Such studies tended to consider context within the narrative of diabetes control in their region. 32 studies described the national context in which the study took place [25, 27, 31, 33, 42, 43, 48, 50, 51, 54, 59–62, 66, 67, 74, 80–82, 85, 86, 91, 92, 96, 98, 100, 104, 111, 112, 115, 116]. This typically involved descriptive statistics to indicate the magnitude and urgency of diabetes as a national challenge, highlighting incidence or prevalence rates and cost burden. These descriptions ranged from brief summaries to comprehensive elaborations. 16 studies described the health system context in which the study took place [32, 35, 46, 58, 69, 75, 78, 88, 93, 94, 102, 106–110]. The contexts described ranged from broad and general to in-depth and extensive.

10 studies considered the context of the population studied or the specific intervention. Examples included the role of healthcare professionals involved in the intervention [38, 77], descriptions of demographic context (e.g. low-income, indigenous, Hispanic) in which an intervention took place [24, 41, 71, 76, 79, 113, 114], and existing structures in which interventions occurred (e.g. financing of prescription medications in the Veterans Affairs healthcare system) [56]. Table 1 provides examples of context considerations in included studies.

**Table 1:** Examples of context considerations in included studies.

*Scale*	*Description*	*Example(s)*
**Regional**	Extensive	“There is substantial evidence to support the fact that diabetes is assuming epidemic proportion in many developing countries, including those of Sub-Saharan Africa (SSA). Of the 246 million estimated global population with diabetes in 2007, 10.7 million resided in Sub-Saharan Africa. This number will increase by 80% to reach 18.7 million by the year 2025. Type 2 diabetes in developing countries and those of Africa is characterized by a high proportion of undiagnosed patients, reaching 80% in some settings. The insidious nature of type 2 diabetes and the low availability and less accessibility of the African population to healthcare contribute to this situation. The consequences of late diagnosis are that most patients in Africa tend to present with chronic complications at diagnosis.”[99]
**National**	Extensive	“Diabetes is emerging as a major clinical and public health concern among the Kuwaiti population. The reported prevalence rate of known type 2 diabetes in 1990 was 7.6%, ranging from 5.6% to 10% in different governorates. In 1996, the overall prevalence rate of type 2 diabetes in Kuwaiti adults of age 20 years and over was as high as 14.8%. A remarkable increase in prevalence has been reported in more recent studies. In one study utilizing a cross-sectional household survey of 2,487 Kuwaiti nationals aged 50 years and over in 2005/2006 from two health governorates, the prevalence of physician-diagnosed diabetes was found to be 50.6%. Type 2 diabetes was detected even in adolescents, according to a population study of Kuwaiti school children, making the disease a public health problem. The burden of diabetes in Kuwait is high, and it has a serious impact on morbidity and mortality.”[112]
	Brief	“Like in many other countries, chronic care tasks are increasingly being delegated from general practitioners (GPs) to nurses in Danish general practices”[67] “in the United States alone the total financial cost attributable to diabetes was estimated at $132 billion”[31]
**Health System**	Brief	“[Christiana Care Health System] CCHS is the largest health care provider and the largest private employer in Delaware. CCHS is self-insured and, like most large companies, has experienced rapid growth in health care expenditures over the past decade.” [74]
	Extensive	“The Medicare Part D program, introduced on January 1, 2006, provides prescription drug coverage for Medicare beneficiaries. One unique feature of the Part D benefit design is the coverage gap (or donut hole). The defined standard benefit in 2008 started with a $275 deductible and a 25% copayment for drug spending between $275 and $2510. After the initial coverage period, beneficiaries entered a coverage gap, in which they paid 100% of the drug cost, until their true out-of-pocket drug spending reached the catastrophic limit of $4050 (or total drug spending of $5726.25). Under the catastrophic coverage, beneficiaries pay the greater of a 5% or a $2.25/$5.60 (generic/brand-name) copayment.” [75]
**Context of direct population studied**	Role of healthcare professionals	“Like many health systems nationally, the Veterans Health Administration (VA) is undergoing a major transformation of primary care to team-based care, by implementing a patient centered medical home (PCMH) model system wide to improve access, coordination, and continuity of care. Pharmacists have been recommended as a standard component of patient-centered medical homes, but their impact on OHA adherence has not been studied. Pharmacists in VA primary care clinics may have a clinically oriented role by providing counseling and education to patients taking diabetes medications. However, pharmacists in VA may also be limited to a purely dispensing role or simultaneously manage both clinical and dispensing tasks.”[77]
	Demographic	“Indigenous Australians have the highest prevalence and incidence of diabetes in Australia and also suffer high rates of preventable complications. Many of these complications can be prevented with better primary care level management however access to culturally appropriate high quality diabetes care is not always evident, especially in remote settings where there is high turnover of health staff. Australian Indigenous adults with type 2 diabetes are on average 10 years younger, have poor glycemic control and lower levels of preventive service up-take compared to non-Indigenous adults with diabetes in a national sample”[41]
	Financing structures	“Veterans Health Administration (VA) medical centers offer more comprehensive medication coverage than almost any other public or private payer in the United States. Drugs on the VA formulary are 100% covered for patients with low incomes or service-connected disabilities. Other VA patients pay a $7 copayment for a 30-day supply of medication treating a nonservice-connected condition. VA patients have no cap on either the total cost of their covered drugs or the number of prescriptions they can fill in a given period, and patients who incur $840 or more in copayment costs during a given year have all subsequent copayments waived.”[56]

### Effect of health system inputs on diabetes outcomes

37 studies explored the impact of health system inputs on diabetes outcomes. The analysis explores studies that had a focus on one type of resource, and complex interventions involving studies with more than one type of resource or system building block. Table 2 summarises the findings of included studies exploring the associations between health systems inputs and diabetes outcomes.

**Table 2:** Summary of findings of studies examining the associations between health systems inputs and T2DM outcomes.

Health System Arrangement	Study	Setting and Sample Size	Study Design	Findings (95% CIs Given in Brackets Where Available)	Risk of Bias Assessment
**Physical Resources**					
**Distance to health facility**	Littenberg. 2006	USA–Patients managed in Primary Care N = 781	Cross-sectional	• OR 0.97 (0.95–0.99) for insulin use per km of driving distance	Low risk of bias
**Human Resources**					
**Pharmacists**					
**Follow up intervention with pharmacist**	Bello et al. 2012	Nigeria–Patients in a primary health facility N = 170	Pre-post	• Mean A1C reduced from 8.08 pre-intervention to 7.08 post-intervention (p<0.001)	Medium risk of bias
**Pharmacist-patient clinic visits including medication review, performing targeted physical assessment, education, reviewing patient medication therapy**	Jacobs et al. 2012	USA- Patients in primary care N = 2121	Randomised controlled trial	• Greater absolute % change in A1c from baseline for intervention group than control group who received usual care directed by their physicians (-0.18 vs -0.8%) (p<0.05)	High risk of bias.
**Pharmaceutical care initiative (Medical record review, pharmacotherapeutic evaluation and patient medication education and monitoring**	Taylor et al. 2003	USA-Patients from areas of severe poverty, low insurance coverage and poor health indicators going to community based family clinics N = 69	Randomised controlled trial	• At 12 months, intervention-group patients more likely than control patients to achieve blood pressure (BP) targets (intervention 91.7% vs. control 27.6%, p = 0.001) • At 12 months, increase from baseline in the percentage of patients at BP (12.5% vs 91.7%, p<0.001), lipid (10.5% vs 77.8%, p<0.001) goals in intervention group • Control group received standard medical care.	High risk of bias
**Outpatient clinical pharmacist programme (face to face consult with patients, decision support tool for medication prescription)**	Spence et al. 2014	USA- Kaiser permanente health system N = 2957	Cohort	• Mean HbA1c in intervention group lower than usual care group (8.48 vs. 8.80, P = 0.024) • Reduction in HbA1c from baseline (-1.25 vs. -0.75, P = 0.001) • 53.5% of intervention group adherent to diabetes medications after 1 year, compared with 37.4% in the usual care group (P = 0.001) • Intervention group saw increase in medication possession ratio (MPR) from baseline compared with usual care group (0.19 vs. 0.15, P = 0.024 • Intervention patients less likely to discontinue diabetes medications (11.7% vs. 35.5%, P < 0.001) and more likely to have their medication prescription filled within 30 days after the end of their supply of the last prescription post-first consultation date (34.8% vs. 12.9%, P< 0.001) • Average days to first medication prescription filled after first consultation date was 79.3 for the intervention group compared with 156.3 for the usual care group (P< 0.001)	Medium risk of bias
**Pharmacist-delivered, community pharmacy-based diabetes care and management programme, including support for blood glucose self-monitoring, education, adherence support, and reminders of checks for complications**	Krass et al. 2007	Australia- patients collecting medication in community pharmacies N = 289	Randomised controlled trial	• Mean reduction in HbA1c in the intervention group was -0.97% (-0.8, -1.14) compared with -0.27% (-0.15,-0.39) in the control group who received usual care from pharmacists (p<0.01)	High risk of bias
**Pharmacist allowed to adjust medication and met with patient to evaluate patient therapy needs and to develop a patient care plan**	Ko et al. 2016	USA–patients in an integrated health maintenance organization N = 150	Pre-post	• Intervention group had lower mean A1c readings compared with control after 1 year (8.18% vs 8.69%, p = 0.012) and 2 years (8.06% vs 8.67%, p = 0.014) • 2-year average decrease in A1c was larger in intervention group compared with usual care group (-1.24 vs -0.59, p = 0.009)	Low risk of bias
**Pharmacist directly involved in patient care and management of patient requiring insulin therapy**	Coast-Senior et al. 1998	USA- patients on insulin therapy in primary care clinics N = 23	Pre-post	• Decreased mean HbA1c concentrations from 11.1% to 8.9% (p = 0.00004)	Medium risk of bias
**Pharmacist intervention (education session and information leaflet distribution)**	Vella et al. 2013	Malta- Pharmacies in Malta N = 30	Pre-post	• Improvement from 24 patients reporting rarely missing a dose of medication pre-intervention to 8 patients post-intervention • Pre-intervention 1 patient reported to "never miss a dose" of medication"; increased to 22 post-intervention	Medium risk of bias
**Pharmacist led shared medical appointments program which included an educational component**	Cohen et al. 2011	USA- patients in a veteran’s affairs medical centre N = 99	Randomised controlled trial	• Compared to standard primary care, treatment group had significant reductions in A1C (-0.41; 95% CI-0.74 to -0.07%, p<0.05). • Compared to standard primary care, treatment group had higher adjusted odds of achieving A1C goals (aOR, 2.73; 95% CI 1.03 to 7.16, p<0.05) and SBP goals (adjusted odds ratio, 3.06; 95% CI, 1.31 to 7.16, p<0.05)	High risk of bias
**Pharmacist-led, patient-centred pharmacotherapy management program (interdisciplinary approach to patients with complex disease state and medication burdens, consultation with a pharmacist where patients were instruced on blood glucose monitoring an dietary practices)**	Monte et al. 2009	USA- Patients in regional primary care group N = 50	Pre-post	• At 6 months and 12 months, A1C and fasting plasma glucose (FPG) reduced compared to baseline (6 months: A1C, -1.1%, p<0.0001 and FPG, -39 mg/dL, p = 0.003. 12 months: A1C -1.1%, p<0.0001and FPG -35 mg/dL, p = 0.005)	Medium risk of bias
**Integration of pharmacist practice into patient-centred medical home**	Berdine et al. 2012	USA- Patients in a medical home N = 200	Pre-post	• Mean A1C decreased from baseline at 1 year (8.3% to 7.7%, p<0.0001) and 2 years (8.25% to 8.10%, p = 0.006)	Medium risk of bias
**Addition of pharmacist to primary care team**	Simpson et al., 2011	Canada- Primary care clinics in a primary care network N = 260	Randomised controlled trial	• Over 1 year, reduction in SBP for intervention patients (-7.4mmHg, 95%CI 4.6–10.2, p<0.001) but no significant reduction in control patients who received usual care by primary care team • Between-group difference in SBP at 1 year in favour of intervention (4.9mmHg, 95%CI 1.0–8.7, p = 0.01) • OR 2.55 (95%CI 1.3–5.01, p = 0.0065) for 10% decrease in SBP	Medium risk of bias
**Face to face meeting with community pharmacists every 3 months which consisted of diabetes counselling**	Oyetayo et al. 2011	USA–Hispanic patients seeking care in a pharmacy network N = 126	Cohort	• Reductions in FPG (163 vs. 149 mg/dL, p = 0.019), SBP (87 vs. 85 mmHg, p = 0.003), and triglycerides (191 vs. 176 mmHg, p = 0.003) • Subgroup analyses of patients with poorly controlled diabetes at baseline revealed reductions in mean HbA1c (9.2% vs 8.6%, p = 0.006), SBP (147 vs. 143 mmHg, p = 0.031), DBP (91 vs. 87 mmHg, p<0.001), triglycerides (259 vs. 219 mg/dL, p<0.001), LDL-C (139 vs. 123 mmHg, p<0.001), and total cholesterol (237 vs. 222 mmHg, p = 0.008)	High risk of bias
**Pharmacists trained in motivational interviewing on patient-centred approaches to achieving health goals, working with patients on intervention teams in person or over the phone**	Heisler et al. 2012	USA- patients in outpatient primary care clinics N = 4622	Cluster randomised pragmatic trial	• Mean SBPs of intervention group decreased to a larger extent as compared to the control group, who received usual care, after intervention (difference of -2.4mmHg, 95%CI -3.4–-1.5, p<0.001)	High risk of bias
**Patients seen by clinical pharmacy specialists for diabetes management**	Quinones et al. 2016	USA- patients using clinical pharmacist services in large urban healthcare system N = 915	Record/ chart review	• Average HbA1c difference of -2.6% from initial to final visit (2010–2013, all p<0.01) • Increase in patients with DM achieving HbA1c >7% 2.1% in 2010, 2.7% in 2011 and 2012, and 3.1% in 2014 (all p<0.01) • Overall increases in absolute proportion of medication adherence at time of discharge: 42.8% in 2010, 43.5% in 2011, 42.8% in 2012, 49% in 2013	Low risk of bias
**Pharmacist meetings to initate diabetes care plan, alongside visits and telephone calls to facilitate diabetes management**	Odegard et al. 2005	USA- patients managed in Primary care N = 77	Randomised controlled trial	• Mean HbA1c in both intervention and usual care groups decreased from baseline 6 and 12 months (p = 0.001), but intervention groups achieved HbA1c decreases with fewer physician visits	High risk of bias
**Presence of pharmacists in clinics**	Kocarnik et al. 2012	USA–Patients managed in Veteran’s Affairs health system N = 280603	Cohort	• No statistically significant effect of pharmacist presence on patients’ medication adherence	Medium risk of bias
**Nurses**					
**Empowering nurses with no previous experience in management of chronic diseases to be directly in charge of running the clinics**	Kengne et al. 2009	Cameroon- T2DM patients N = 225	Pre-post	• 1.6mmol/L difference in mean FPG levels between baseline and final visit (95% CI: 0.8–2.3, p<0.001)	Low risk of bias
**Transferring diabetes care to nurses specialized in diabetes in secondary care**	Houweling et al. 2009	Netherlands- Patients managed in general practice N = 93	Randomised controlled trial	• After 1 year, more intervention group patients (2.2% to 33.3%, p<0.002) achieved target HbA1c <7% compared to control group who received standard care (10. 5% to 26.3%) •	High risk of bias
**Involvement of nurses in diabetes care in general practice**	Juul et al. 2012	Denmark–Patients in general practice N = 258	Cross-sectional	• Proportion of patients with HbA1c ≥8 was -3.7% (95%CI -6.7 to -0.6%) between practices with well-implemented nurse-led diabetes consultations compared with no nurses employed (p<0.05)	Low risk of bias
**Nurse practitioners in collaborative practices with primary care clinicians in helping improve control of HbA1c, BP, and LDL-C in adults with uncontrolled hyperglycemia**	Richardson et al. 2014	USA- Patients in 2 ambulatory care internal medicine modules in the Kaiser permanente health system N = 28	Record/ chart review	• Post-intervention, 13 patients (50%) achieved HbA1c <8% compared to pre-intervention (0 patients) (p<0.001)	Medium risk of bias
**Transferring diabetes management to practice nurses in primary care setting**	Houweling et al 2011	Netherlands- patients managed in general practice	Randomised controlled trial	• No significant between-group differences with respect to reduction in HbA1c, blood pressure and lipid profile • Control group received conventional care from general practitioners	High risk of bias
**Involvement of non-ICU nurses, providing specific training an obligatory certifation, and independent authority of all certified nurses to initate the correctional insulin algorithm in patients with diabetes**	Manders et al. 2016	Netherlands- patient admitted to a medical centre N = 410	Trial	• No significant differences in mean blood glucose and FPG levels between intervention and control groups • Control group was derived from patients hospitalised prior to implementation of the intervention.	High risk of bias
**Nurse behavioural management of diabetes and hypertension in community practices**	Edelman et al. 2015	USA- patients in primary care N = 377	Randomised controlled trial	• No A1c and SBP differences between intervention group compared to control group, who received calls which were not tailored and discussed topics not relevant to diabetes or hypertension management. •	Medium risk of bias
**Clinic nurse practitioners had visits to a diabetes specialist, conference calls, guidance by faxing flow sheets to specialist and 24 hour reponse to critique treatment plan**	King et al. 2009	USA- Patients in primary care N = 135	Randomised controlled trial	• No significant reduction in HbA1c from baseline comparing treatment vs. control group • The control group was not contacted, and only their charts were reviewed by the research team.	High risk of bias
**Community health workers**					
**Community health workers delivery diabetes curriculum targeting barriers to diabetes management**	Collinsworth et al. 2013	USA–diabetes self-management program for uninsured and underserved patients N = 497	Mixed methods	• Improved mean A1C value from 8.7% at to 7.4% following participation (p<0.001) • Improved mean SBP (baseline 129.8mmHg vs. 1-year follow-up 127.3, p = 0.03)	High risk of bias
**Recruited an indigenous health worker resident in the community to work as part of the primary care team, they were trained on clinical aspects of diabetes and other chronic conditions, and how to manage diabetes.**	McDermott et al. 2015	Australia- poorly controlled patients with diabetes in indigenous communities managed in primary care N = 213	Randomised controlled trial	• At 18 months follow-up, HbA1c reduction in the intervention group (10.8% to 9.8%) was greater than reduction in control group (10.6% to 10.3%), p = 0.0018 • The control group was placed on an intervention waitlist, and given the intervention after the study.	Medium risk of bias
**Community health workers compared to case management and usual care**	Babamoto et al. 2009	USA–Hispanic patients managed in family health centres N = 318	Randomised controlled trial	• Mean A1C decreased from 8.6% to 7.2% (p<0.05) in the community health worker group, 8.5% to 7.4% (p<0.05) in the case management group, 9.5% to 7.4% (p<0.05) in the standard provider care group • Proportion of patients who reported never forgetting to take their medication decreased in case management (77%to 55%, p <0.05) and standard provider care groups (67% to 50%, p < 0.05) • Proportion of patients reporting never forgetting to take their medication decreased in case management (77%to 55%, p <0.05) and standard provider care groups (67% to 50%, p <0.05)	High risk of bias
**Peers**					
**Reciprocal peer support programme conducted (i.e. an intervention at encouraged patients receive and provide support to one another)**	Heisler et al. 2010	USA- Veterans managed in nurse care management N = 244	Randomised controlled trial	• Mean HbA1c levels in intervention group reduced (-0.29%); mean HbA1c levels control (nurse case management) group increased (0.29%) (between-group difference 0.58%, p = 0.004) • Among patients with baseline HbA1c >8.0%, intervention group had a mean decrease of 0.88%, compared with a 0.07% decrease in control group (between-group difference 0.81%, p<0.001)	Low risk of bias
**2-year peer support intervention (included recruitment and training of peer supporters, nine group meetings led by peer supporters in participant’s own general practice, and a retention plan for peer supporters)**	Smith et al. 2011	Ireland- Patients in general practice N = 395	Cluster randomised controlled trial	• At two-year follow-up, no significant differences in HbA1c, SBP, total cholesterol despite trend towards decreases in proportion of patients with poorly controlled risk factors at follow-up	Low risk of bias
**Physicians**					
**Regular physician for diabetes care**	Hueston et al. 2010	USA- patients at a family medicine centre of a university hospital N = 705	Record/ chart review	• Patients with a regular provider had lower average HbA1C (7.7 vs. 8.5, P = 0.01) compared to patients without	Medium risk of bias
**GP screening activity**	Spigt et al. 2009	Netherlands- Primary care centres in a university network N = 58919	Record/ chart review	• Patients with diabetes in primary care had worse HbA1c than patients in secondary/tertiary care (pri care 8.4 ± 1.8% vs. sec/ter care 8.1 ± 1.6%, p < 0.001) • Patients with diabetes treated in primary care setting had better BP control than those in secondary/tertiary setting (BP target 140/90 mmHg, pri care 42.3% vs. sec/ter 46.6%, p < 0.05)	Low risk of bias
**Clinical inertia (inadequate intensification of therapy by the provider)**	Ziemer et al. 2005	USA- Diabetes clinic N = 2157	Cohort	• Tendency of individual providers to intensify therapy associated with lower HbA1C levels (P < 0.0001) • 10% higher frequency of intensification associated with a 0.15% lower level of A1C • A single episode of intensification of therapy associated with an average 0.7% A1C reduction	Low risk of bias
**Physician certification**	Pinsky et al. 2011	USA- Nationwide data N = 8011	Record/ chart review	• Patients managed by certified physicians (certification recognises physicians and practices providing high-quality diabetes care) more likely to receive prescriptions for oral antihyperglycemic agents than those managed by non-certified physicians (mean prescriptions per patient per month 0.49 vs. 0.46, p = 0.02)	Medium risk of bias
**Vocational registration of GPs**	Kamien. 1994	Australia–audit data of GP practitioners and patients N = 671	Cross-Sectional	• No difference in HbA1c between vocationally registered and non-vocationally registered doctors	Medium risk of bias
**Patient- physician gender concordance**	Schmittdiel et al. 2009	USA- Kaiser Permanente North California N = 157458	Cross-Sectional	• Female patients of female physicians most likely to have HbA1c<8% (70% vs. 66%–68%) • Patient and physician gender interaction associated with significant LDL-C control, with male patients of male physicians having the highest proportion of patients at or below target	Low risk of bias

#### Physical resources

One cross-sectional study from the United States found an association between driving distance from primary care facility and likelihood of insulin use (odds ratio (OR) for using insulin associated with each kilometre of driving distance, 0.97, 95% confidence interval (CI) 0.95, 0.99; p = 0.01) [68]. Living within 10km of a primary care facility was associated with increased likelihood of insulin use (OR 2.29, 95%CI 1.35–3.88; p = 0.02).

#### Human resources

36 studies examined the effect of human resource inputs on diabetes outcomes.

Pharmacists: 17 quantitative studies explored the impact of pharmacists on diabetes outcomes. All but one [88] took place in high-income countries. 11 found positive effects of pharmacist care on diabetes control and adherence outcomes.

Seven studies from high-income countries reported positive impacts of pharmacists administering patient care. Two trials, both conducted in the United States, obtained positive results. One found a significantly greater absolute percent decrease in HbA1c from baseline among those seeing a pharmacist [30]. The other, in university owned neighbourhood clinics, reported that the intervention group achieved reductions in HbA1c with fewer physician visits compared to patients receiving usual care [33].

A cohort study in the United States reported a lower mean HbA1c among those in an outpatient programme involving face-to-face pharmacist consults (p = 0.024), and significantly reduced from baseline [76]. Although there was no significant between-group difference in mean medication possession ratio (MPR), intervention patients saw an increase in MPR from baseline (p = 0.024). Patients receiving the intervention were less likely to discontinue diabetes medications (p<0.001) and more likely to have their medication prescription 30 days after the end of their supply of the last prescription following their first consultation date (p< 0.001).

An Australian randomised controlled trial found that a pharmacist-delivered community-based care and management programme was associated with a mean decrease in blood glucose levels over six months [32]. Improvements in HbA1c were greater in the intervention group (-0.97% (95% CI: -0.8, -1.14, p<0.01) compared to controls (-0.27% (95% CI: -0.15, -0.39, p<0.01)).

A pre-post study from the United States found that an intervention with pharmacists adjusting medications, evaluating therapeutic needs, and developing care plans recorded significantly lower mean HbA1c readings compared with the control group after one and two years. The two-year average decrease in HbA1c was greater for the intervention group compared to the control (p = 0.009) [100].

Another pre-post study from the United States found that involving clinical pharmacists in direct patient care of insulin-dependent patients in primary care led to a significant decrease in mean HbA1c post-intervention [94]. A Maltese pre-post study found that an education-focused pharmacist intervention was associated with a smaller proportion of patients “rarely” missing a dose of medication and a larger proportion of patients reporting “never missing a dose” of medication post-intervention [98].

Four studies reported positive impacts of pharmacist-led patient management. A randomised controlled trial in the United States found that pharmacist-led shared medical appointments was associated with significant reductions in HbA1C (-0.41; 95% CI -0.74 to 0.07) and significantly higher odds of attaining HbA1C goals (adjusted OR 2.73; 95% CI, 1.03 to 7.16) compared to usual care [26]. A pre-post study in the United States found that a pharmacist-led, patient-centred pharmacotherapy management programme was associated with significantly reduced HbA1c and fasting plasma glucose parameters for patients with diabetes who had complex disease and medication burdens at six and 12 months when compared to baseline [91]. A Nigerian pre-post study found reductions in mean HbA1c and fasting blood sugar in those receiving monthly follow up pharmacists over three months at a primary healthcare facility [88]. A pre-post study in the United States found that having a pharmacist practice in a patient-centred medical home (a team-based model of care led by a personal physician who provides continuous and coordinated care throughout a patient’s lifetime to 15 outcomes) was associated with significant decreases in patients’ mean HbA1c from baseline at one- and two-year time points [89].

Four studies, all from North America, found mixed effects of pharmacist involvement in service delivery on diabetes control and adherence outcomes. A Canadian randomised controlled trial [42] found a reduction in systolic blood pressure (SBP) of 7.4mmHg (95%CI 4.6–10.2; p<0.001) over one year in patients managed by a team to which a pharmacist had been added, with a between-group difference in SBP of 4.9mmHg, 95%CI 1.0–8.7; p = 0.01. However, there were no significant changes in glycaemic control and lipid parameters. A pragmatic cluster randomised trial in the United States found that a clinical pharmacist-led outreach programme (working with patients in person or over the phone, motivational interviewing) had only short-term positive effects for primary care patients [46]. Immediately post-intervention, the mean SBP of the pharmacist-led group decreased compared to the control group (-2.4mmHg, 95%CI 1.5–3.4, p<0.001). However, the control group achieved similar results six months post-intervention. A United States cohort study found that a face-to-face community pharmacist intervention focused on counselling and education for Hispanic patients with diabetes led to reductions in fasting plasma glucose (p = 0.019), SBP (p = 0.003), and triglycerides (p = 0.003) from baseline to 12 months, but not in mean HbA1c. Subgroup analyses of patients with poorly controlled diabetes at baseline showed significant reductions in mean HbA1c, SBP, diastolic blood pressure (DBP), and lipids [71]. A record/chart review in the United States found that diabetes management led by clinical pharmacists [102] was associated with a reduction in mean HbA1c and an increase in patients achieving HbA1c <7% over four years. However, no significant improvements were found in SBP, DBP, lipid measures, or medication adherence.

Two studies found no impact of pharmacists on outcomes. A randomised controlled trial in the United States exploring the impact of a pharmacist intervention (including meetings and phone-calls to initiate care plans) on poorly-controlled patients with diabetes managed in primary care found no difference in mean HbA1c or self-reported medication adherence [33]. A cohort study in the United States found no significant effect on medication adherence associated with the presence of a pharmacist in Veterans Affairs clinics [77].

Nurses: Eight studies, all quantitative, looked at the impact of nurses on diabetes control and adherence outcomes. All but one [99] took place in high-income countries. Three studies found positive impacts on control of service delivery by nurses. A Dutch randomised controlled trial explored the impact of transferring routine aspects of diabetes care in hospital outpatient clinics to diabetes specialist nurses [29]. After one year, significantly more patients receiving care from nurses achieved HbA1c <7% compared to the control group. A Cameroonian pre-post study [99] found a significant reduction in mean fasting blood glucose (FBG) in non-insulin-dependent patients’ following a nurse care empowerment scheme. A Danish cross-sectional study [67] found that the proportion of patients with HbA1c ≥8% significantly decreased in general practices with well-implemented nurse-led diabetes consultations, compared to practices without.

One record/chart review in the United States reported mixed results. It evaluated the impact of incorporating nurse practitioners into collaborative practices with primary care clinicians [106] and found that, post-intervention, 50% of patients achieved HbA1c <8% compared to 0% of patients pre-intervention (p<0.001). There were no significant changes in the proportion of patients achieving BP and cholesterol targets.

Four studies, all trials, reported no significant effects of service delivery by nurses on diabetes control. Two were conducted in the Netherlands and examined the impact of diabetes management by nurses [27] and nurse-driven, protocol-based correctional therapy on patients [50]. Two were conducted in the United States and explored the effect of nurse-led behavioural management [37] and empowerment of nurse practitioners to provide comprehensive patient care [31].

Physicians: Six studies, all quantitative and conducted in high-income countries, investigated the impact of physicians on diabetes outcomes. A cohort study in the United States found that 10% increased frequency of therapeutic intensification (i.e. increasing the dosage or amount of hypoglycemic medication a patient takes) by a physician was associated with a 0.15% lower level of HbA1c (p<0.0001) among patients in an urban health system. A single episode of therapeutic intensification was associated with an average 0.7% reduction in HbA1c (p<0.001) [85].

A record/chart review in the United States reported that newly-diagnosed patients with diabetes at a family medicine clinic of a university hospital with a regular physician reported lower mean HbA1c values than patients without a regular physician [103]. A Dutch record/chart review found that symptom-catalysed (encouraged by the onset of symptoms in the patient), opportunistic, or patient-requested general practitoner (GP) screening activity was significantly related to the presence of a diabetes diagnosis [101].

A review of United States claims records found a positive association between physicians’ certification by a national-level quality assurance organisation and patient adherence [105]. Patients managed by certified physicians were more likely to receive oral anti-hyperglycaemic drug prescriptions (mean prescriptions per patient per month 0.49 vs. 0.46, p = 0.02).

An Australian cross-sectional study compared the impact of vocationally-registered vs. non-vocationally-registered general practitioners (GPs) on diabetes control [59]. Vocational registration entails the enrolment of GPs as part of improving professional standards, rewarding high-quality practice, and enabling GPs’ access to higher rebates in the publicly-funded universal healthcare system. It found no difference in mean HbA1c. A United States cross-sectional study [70] found an association between patient-physician gender concordance and diabetes control, with female patients of female physicians most likely to have HbA1c <8%. However, this was not due to differences in medication adherence.

Community health workers: Three studies focused on the impact of community health workers (CHWs). Two reported positive impacts on diabetes outcomes. A ixed-methods study in the United States found that a culturally-relevant diabetes self-management education programme led by trained, bilingual CHW to improved mean HbA1c and SBP among uninsured and underserved Hispanic patients [114]. An Australian trial found that poorly-controlled indigenous patients managed in primary care and receiving a clinical, management, education, and social and family support-focused CHW intervention by a trained indigenous CHW resident in the community had improved mean HbA1c levels compared to controls [41]. Meanwhile, a randomised trial of Hispanic/Latino patients with diabetes in the United States managed at family health centres found no differences in control and adherence between patients receiving care delivered by full-time, trained bilingual CHWs who had T2DM/had experienced it via a family member or friend vs. case management and standard care [24].

Peers: Two trials investigated the impact of peers on diabetes outcomes. Results were mixed. A randomised controlled trial in the United States of veterans with poor glycaemic control receiving usual nurse care vs. reciprocal peer support (i.e. an intervention that encouraged patients with diabetes to receive and provide support to each other) [44] found that peer support significantly impro17ved mean HbA1c levels while in those receiving nurse-led care mean HbA1c levels increased. Among patients with baseline HbA1c <8.0%, patients receiving peer support (-0.88%) demonstrated greater improvement in mean HbA1c (-0.07%) compared to those receiving nurse care. Meanwhile, an Irish cluster randomised controlled trial compared outcomes of patients receiving standardised diabetes care vs. a two-year peer-support intervention [45]. At two-year follow-up, there were no significant differences in HbA1c, SBP, and total cholesterol.

#### Intellectual resources

No included studies evaluated the relationship between intellectual resources, such as use of guidelines, and diabetes outcomes.

#### Social resources

No included studies evaluated the effects of social resources on diabetes outcomes.

### Health systems financing

21 studies examined the effect of health systems financing on diabetes outcomes. 15 were quantitative and conducted in high-income countries: 13 in the United States and two in Canada. Six were qualitative, and all but one [111] took place in lower and middle-income countries. The high-income country study [111] was the only study not reporting cost or financial difficulty as a barrier to diabetes control, treatment, or adherence. Table 3 summarises the findings of included studies exploring the associations between healthcare financing and diabetes outcomes.

**Table 3:** Summary of findings of studies examining the associations between healthcare financing and T2DM outcomes.

Health System Arrangement	Study	Setting and Sample Size	Study Design	Findings (95% CIs Given in Brackets Where Available)	Risk of Bias Assessment	
**Health Systems Financing**						
**Cost-sharing on outcomes**	Gibson et al. 2010	USA- patients in a healthcare system with employer-sponsored benefits N = 152090	Cross-Sectional	• For OAD users, the OR for non-adherence as prescription drug cost sharing increased was 0.974 (0.970–0.984, p<0.1); for OAD-only users, the OR was 0.978 (0.973–0.984, p<0.01) • For all OAD users, the OR for non-adherence with increasing cost-sharing for physician visits was 0.996 (0.994–0.997 p<0.01); for OAD-only users the OR was 0.995 (0.993–0.996, p<0.01)	Low risk of bias	
**Cost-sharing on outcomes**	Hsu et al. 2006	USA- Medicare+ choice beneficiaries in a Kaiser permenente health system N = 199179	Cohort	• For subjects with capped benefits, OR for non-adherence to antidiabetic drugs was 1.33 (1.18–1.48) • OR for elevated glycated hemoglobin was 1.23 (1.03–1.46)	Low risk of bias	
**Cost-sharing on outcomes**	Hunt et al. 2009	USA- patients enrolled in a commercial exclusive provider organization plan having different cost-sharing amounts N = 5189	Cohort	• For each $5 increase in cost share, 0.1 increase in HbA1c (p = 0.02) • For every increase in patient cost share by $1, 1.2% reduction in odds of oral diabetic medication adherence (p<0.0001)	Low risk of bias	
**Cost-sharing on outcomes**	Ngo-Metzger et al. 2012	USA- ethnically diverse patients in various outpatient clinics N = 1361	Cross-sectional	• Perceived financial burden as associated with HbA1c ≥8% (aOR = 1.7, 95%CI 1.09–2.63) • Being uninsured (aOR = 1.9, 95%CI 1.13–3.21) and non-adherence (aOR = 1.49, 95%CI 1.06–2.08) associated with HbA1c	Medium risk of bias	
**Cost-sharing on outcomes**	Elliott et al. 2013	USA- patients in a private health system N = 242	Cohort	• Between baseline and follow-up, no significant changes in glycemic control • Participants were more likely to self-report being adherent to oral diabetes medications at 1-year follow-up (p = 0.011).	Medium risk of bias	
**Insurance status on outcomes**	Grogan et al. 2010	USA- participants in the bypass Angioplasty Revascularization Investigation 2 diabetes trial N = 776	Cross-Sectional	• Compared to patients with private or no insurance, patients with public insurance have lower mean A1C (private 8.2 vs. public 7.7 vs uninsured 8.29, p<0.001) and lower proportion of patients with A1C ≥7% (private 71.6% vs. public 61.2% vs. uninsured 68.3%, p = 0.001)	Low risk of bias	
**Insurance status on outcomes**	Piette et al. 2004	USA- Veteran affairs health systems N = 766	Cross-Sectional	• Patients with private insurance almost twice as likely to report underusing medication in the prior 12 months as VA patients (P <0.0001) • Patients who reported cost-related medication underuse had an average HbA1c of 8.7% (p<0.001) • Cost-related medication underuse associated with 0.6% (0.2–0.9) absolute increase in HbA1c levels (p = 0.005)	High risk of bias	
**Insurance status on outcomes**	Tan et al. 2015	USA- Nationwide data N = 452383	Cross-Sectional	• Diabetes control was highest at 68.9% for commercially insured patients (69.1–68.7) than 53.7% for Medicare (53.5–54.0) and 52.7% for Medicaid patients (52.3–53.0) (p<0.05) • Average PDC and drug adherence were higher at 83% (82.9–83.1) for patients insured by Medicare than 76.6% (76.5–76.8) for patients who were commercially insured and 74.4% for Medicaid insured (74.2–74.6) (p<0.05)	Medium risk of bias	
**Socioeconomic factors on healthcare seeking behaviour of newly diagnosed persons with T2DM**	Burge et al. 2000	USA-community-wide diabetes screening programme N = 118	Cross-Sectional	• Lack of insurance coverage as primary reason that patients with newly diagnosed diabetes fail to seek medical care (p<0.001)	High risk of bias
**Extent of insurance coverage and outcomes**	Soumerai et al. 2004	USA- patients of a health management organization N = 3219	Time-series	• Initiation of self-monitoring (as a result of financial coverage) not associated with improved HbA1c levels in those with good or adequate baseline glycemic control • Among those with poor glycemic control, initiators of self-monitoring lowered their mean HbA1c level by 0.63% compared with noninitiators (1.14–0.12, p = 0.03) • Compared with noninitiators of self-monitoring, initiators had improvements in regularity of medication use by 6 months after initiation: −19.5 days between dispensings among those with low refill regularity (27.7−11.3); −9.7 days among those with moderate regularity (12.3−7.1), and mean HbA1c level reduced by 0.63% (1.14% -0.12%) • Among those with moderate refill regularity by 6 months after initiation of self-monitoring, initiators reduced mean gaps between dispensings by 9.7 days compared with noninitiators (12.3−7.1) • Among those with low baseline refill regularity, initiators of self-monitoring had immediate reductions in mean gaps of 19.5 days compared with noninitiators (27.7−11.3)	Medium risk of bias	
**Extent of insurance coverage and outcomes**	Bowker et al. 2004	Canada- patients managed in pharmacies N = 405	Cross-Sectional	• Patients with insurance had lower HbA1c than patients without insurance (7.1 vs. 7.4, p = 0.03) • Patients with insurance for testing strips had significantly lower HbA1C concentrations (adjusted difference 0.5%, p = 0.006) than patients without insurance	High risk of bias	
**Extent of insurance coverage and outcomes**	Johnson et al. 2006	Canada–patients without private insurance and not using insulin N = 458	Randomised Controlled Trial	• Reducing financial barriers by providing free testing strips did not significantly improve glycaemic control in patients	Medium risk of bias	
**Extent of insurance coverage and outcomes**	Gu et al. 2010	USA- prescription drug claims data by national pharmacy benefit management company N = 12881	Cohort	• Patients with no coverage in the Medicare Part D coverage gap had a 38% reduction (OR = 0.617, P<0.0001, 95% CI = 0.523, 0.728) in odds of being adherent after reaching the Medicare Part D coverage gap, compared with patients with full coverage • Patients with only generic coverage in the Medicare Part D coverage gap had a 30% reduction (OR = 0.702, P<0.001,95% CI = 0.604, 0.816) in odds of being adherent after reaching the Medicare Part D coverage gap, compared with patients with full coverage	Medium risk of bias	
**Extent of insurance coverage and outcomes**	Patel et al. 2006	USA- Patients in an outpatient medical assistance programme N = 143	Pre-post	• 0.85% reduction in HbA1c (0.34–1.37, p = 0.002) • 33% increase in patients who achieved HbA1c <7% (p = 0.008) • Total cholesterol decreased by 25.7mg/dl (11.1–40.2. p = 0.001)	Medium risk of bias	
**Extent of insurance coverage and outcomes**	Pawaskar et al. 2010	USA- state level Medicaid patients N = 8581	Cohort	• Patients in capitated plans had 5% lower mean oral antidiabetic medication adherence than those in fee for service plans (p<0.05) • Odds ratio of adherence among patients in capitated health plans was 0.89 (0.82–0.98, p = 0.05) of medication adherence compared to patients in fee for service plans	Low risk of bias	
**Barriers to achieving diabetes self-management**	Gazmararian et al. 2009	USA- economically disadvantaged patients with diabetes N = 35	Qualitative	• Cost not mentioned as a barrier to medication adherence	Low risk of bias	
**Barriers to primary care management**	Alberti et al. 2007	Tunisia- Patients with diabetes and healthcare providers in primary care N = 26	Qualitative	• Patients and health professionals quoted financial reasons as the cause of poor patient compliance (compliance in this study refers to adherence to diet, medications, blood tests and referrals)	Low risk of bias	
**Health system constraints in managing T2DM**	Bhojani et al. 2013	India- Patients with diabetes in an urban slum N = 16	Qualitative	• Financial constraints as major barrier to accessing chronic illness medication that should be taken for years or a lifetime	Low risk of bias	
**Patients perspective of care**	Lewis et al. 2014	Bangledesh- Patients with diabetes managed in various healthcare facilities N = 31	Qualitative	• Access to appropriate diagnosis and subsequent treatment was restricted by availability and costs of services	Medium risk of bias	
**Barriers in medication taking**	Jeragh-Alhaddad et al. 2015	Kuwait- Patients with diabetes managed in GP or hospitals N = 20	Qualitative	• Unavailability of medications, difficulties accessing physicians and medications, inequalities in care provision and medication supply at different healthcare facilities, and lack of trust in the government healthcare system as barriers to medication adherence	Low risk of bias	
**Experiences of diabetes care**	Mendenhall et al. 2015	South Africa- Low income black patients with diabetes N = 27	Qualitative	• Structural barriers, e.g. overcrowded clinics and poor access to medicines, as impeding adherence to treatment	Low risk of bias	

#### Cost-sharing and outcomes

Five studies in the United States examined the relationship between cost-sharing (i.e. when patients pay out-of-pocket for a portion of healthcare costs not covered by health insurance) on outcomes. All studies found that adherence and/or control measures decreased as cost-sharing increased. These findings were consistent across different types of cost-sharing and health schemes [60, 65, 74, 78, 80].

#### Insurance status and outcomes

Four studies looked at the impact of insurance status on outcomes. All were from the United States and cross-sectional. One found that publicly-insured patients had significantly lower mean HbA1c values (p<0.001) and better control (HbA1c ≤7%, p<0.001 than those with private insurance [66]. A study of elderly patients found that privately insured patients were almost twice as likely to report diabetes medication underuse compared to patients in the Veterans Affairs system (p<0.0001) [56]. Another study using national data [62] found the highest diabetes control among commercially-insured patients, compared to those covered by Medicare or Medicaid, but adherence was higher among Medicare beneficiaries. Another explored the impact of socioeconomic factors on control among newly-diagnosed patients with diabetes participating in a community-wide screening programme [53] and found that lack of insurance coverage was predictive of patients failing to seek medical care.

#### Extent of healthcare insurance and outcomes

Six studies looked at the impact of different levels of healthcare insurance coverage (i.e. the extent to which different care services, treatment options, medications, and/or self-monitoring and testing equipment are covered under a healthcare insurance pan) on outcomes. Four found that broader coverage was associated with better outcomes. Two, a time-series study among patients with a health maintenance organisation in the United States [107] and a Canadian cross-sectional study of patients receiving care at community pharmacies [52], found that the provision of free monitors/testing supplies (e.g. blood glucose monitors, insurance coverage for supplies e.g. glucometer strips) improved control.

Two studies, both from the United States, found that increased coverage of drugs was associated with improved adherence. A cohort study examined the relationship between Medicare Part D benefit coverage (a federal government programme to subsidise the cost of prescription drugs and drug insurance premiums for Medicare beneficiaries) [75], finding that beneficiaries without coverage (OR 0.617, P<0.0001, 95% CI 0.523, 0.728) or generic coverage only (OR 0.702, P<0.001,95% CI 0.604, 0.816) were less likely to be adherent than those with full coverage of generic and branded drugs. A pre-post study found that uninsured outpatients participating in a pharmacy-managed medication programme providing free medication [92] had significant reductions in mean HbA1c (p = 0.002) and total cholesterol (p = 0.001), and an increase in proportion of patients achieving HbA1c <7%. However, a Canadian randomised controlled trial of free (versus out of pocket payment) self-monitoring supplies [39], found no difference in six-month HbA1c between intervention and control groups. Only one cohort study examined the relationship between type of healthcare financing plan and adherence among Medicaid patients from across the country [82]. It found that patients in capitated plans had 5% lower mean oral antidiabetic adherence than those in fee-for-service plans (p<0.05).

#### Impact of financial factors on outcomes

Six studies, all qualitative, reported on the impact of financial factors (e.g. cost of services, medication, lifestyle management, and the ability of persons with diabetes to pay for them) on outcomes. A Tunisian study using interviews with patients and healthcare professionals in primary care settings [109] and an Indian study using interviews with patients who have diabetes living in urban slums [110] both found financial factors to be a key barrier to access to medication, affecting adherence to diet, medication, blood tests, and referrals. A Bangladeshi study of interviews with patients who have diabetes managed at various care facilities [108] reported that availability and cost of services impeded access to appropriate diagnosis and subsequent treatment.

A Kuwaiti study using interviews with patients who have diabetes and are on oral medication managed in general practice or hospitals [112] reported unavailability of medications, difficulties accessing physicians and medications, inequalities in care provision and medication supply at different healthcare facilities, and lack of trust in the government healthcare system as barriers to adherence. A South African study interviewed low-income female patients with diabetes [113] and found that patients’ adherence to medication was affected by structural factors in the health system, including overcrowded clinics and poor access to medicines. However, a series of study of focus groups conducted in the United States with economically-disadvantaged urban-dwelling African-American patients with diabetes [111] found that the main contributors to lack of medication adherence were denial of consequences and a lack of understanding of the disease, and not cost or financial concerns.

### Service delivery

26 studies investigated the relationship between health service delivery and diabetes outcomes. Table 4 summarises the findings of studies examining associations between service delivery and diabetes outcomes.

**Table 4:** Summary of findings of studies examining the associations between service delivery and T2DM outcomes.

Health system arrangement	Study	Setting and sample size	Study design	Findings (95% CIs Given in Brackets Where Available)	Risk of Bias Assessment
**Integrated/ innovative models of care**					
**Multifactorial intervention (nutritional-hygienic measures, smoking cessation, and intensification of pharmacologic treatment with physicians following clinical practice guidelines**	Tranche et al. 2005	Spain- Primary care centres N = 3466	Cohort	• Significant results (p<0.001) for baseline vs end point % patients achieving HbA1c target <7.5% (74.9% vs 90.6%), all BP goals (<130/85: 3.5% vs 23.3%, <130/80: 1.8% vs 13.6%, <140/90: 15.2% vs 72.4%), and lipid goals (LDL <130 and HDL >40mg/dl: 5.9% vs 40.9%, triglycerides <200mg/dl: 75.2% vs 89.8%) • Significant results (p<0.001) for all indicators comparing baseline and final visit measurements: SBP (149.7 vs 133), DBP (88.6 vs 79.5), total cholesterol (223.4 vs 202), LDL-C (142.1 vs 124.1), HDL-C (49.9 vs 52.7), triglycerides (158.7 vs 139.4), HbA1c (6.9 vs 6.5)	Low risk of bias
**Structured care at GP supported by allied health, with computerized patient register**	De Sonnaville et al. 1997	Netherlands–patients managed in general practice N = 359	Cohort	• At 2 years, mean HbA1c decreased from 7.4 to 7.0% in structured care patients and rose from 7.4 to 7.6% in usual care patients (p = 0.004) • % patients with HbA1c >8.5% decreased from 21.4% to 11.7% in structured care patients and rose from 23.5% to 27.9% in usual care patients (p = 0.008) • HbA1c <7% achieved in 54.3% of those receiving structured care compared to 44.1% of usual are patients (p = 0.013)	Medium risk of bias
**Structured personal care including quarterly consuiltations and individualized goal setting for risk factors**	Nielsen et al. 2006	Denmark- Patients in primary care settings N = 874	Cluster randomised pragmatic trial	• Median HbA1c level was 8.4% in women receiving structured care vs. 9.2% in women receiving usual care (p<0.001) • Women receiving usual care had HbA1c levels 1.1 times higher than women receiving structured care (1.06–1.14, p<0.001).	High risk of bias
**“Care package” for patient including Motivational interviewing for goal setting by healthcare professional, financial incentives based on network achievement of targets and the formation of GP networks**	Hull et al. 2014	UK- clinical data used in assessing quality improvement in a primary care trust N = 41210	Cohort	• Average HbA1c value of all patients with T2DM fell from 7.80% to 7.66% between 2009 to 2012 • Achievement of cholesterol and BP targets increased from 35.3% to 46.1%	Low risk of bias
**Structured education program based on patient empowerment managed by multidisciplinary team**	Musacchio et al. 2010	Italy-diabetes clinic N = 1004	Pre-post	• % of patients with HbA1c ≤7% increased from 32.7% to 45.8% (p < 0.0001) after 12 months follow-up • % of patients with HbA1c ≥9% decreased from 10.5% to 4.3% (p < 0.0001) after 12 months follow-up • % of patients with LDL-C < 100 mg/dl increased from 39.7% to 47.3% (p < 0.0001) after 12 months follow-up • % of patients with LDL ≥ 130 mg/dl decreased from 26.6% to 19.7% (p<0.001) after 12 months follow-up	Medium risk of bias
**Integrated health management model including health record establishment, health evaluation and health management**	Chao et al. 2015	China- patients receiving care from an endocrinology clinic in a district hospital N = 100	Randomised controlled trial	• Mean FBG in the management group -0.82 mmol/l vs. usual care group +0.06m/mol (p = 0.042)	High risk of bias
**Case management of patients with diabetes in outpatient settings**	Yuan et al. 2016	China- Hospital N = 120	Randomised controlled trial	• HbA1c reduced in CM group compared to control group at 6 months compared to baseline, with least mean of 0.43 (95% CI: 0.83, 0.03, p = 0.034) • Statistically significant reductions did not persist at 12 and 24 months • % of participants with HbA1c ≤7.0% was higher over time in the CM group (45.5% at baseline, 54.5% at 6 months, 60.0% at 12 months, and 61.8% at 24 months) • At 24 months, % of participants with HbA1c 7.0% higher in CM group than in control group (61.8% vs. 41.5%, P = 0.035)	Medium risk of bias
**Continuity of care clinic by general internal medicine specialists**	Chalermsri et al. 2014	Thailand–patients managed at continuity of care clinic N = 757	Case-control	• Mean HbA1c lower in Continuity of care (CC) clinic group vs. Outpatient department (OPD) group (7.3 vs. 7.8, p<0.001) • No. of patients who achieved HbA1c <7% in CC clinic group was 123 (32.1%) vs. 91 (24.3%) in the OPD group (p = 0.039)	Medium risk of bias
**Establishing patient education goals, hiring diabetes nurse care managers and developing clinical practice guidelines to managing risk factors, delivering appropriate pharmacological therapies, conducting regular laboratory evaluations and specialist referral**	Maschak-Carey et al. 1999	USA- patients recently in the emergency department or had been admitted to the hospital for diabetes-related problem N = 1779	Record/ chart review	• Before enrolment, average HbA1c values were 9.03 and fell in study participants to 8.3 (p = 0.03).	Medium risk of bias
**Care management model including a medical record system, training providers and rectifying care gaps**	Bunting et al. 2011	USA- self-insured health plan members N = 149	Pre-post	• % patients achieving HbA1c goals increased from 38% to 53% • % patients achieving LDL-C goal increased from 46% to 67% • % patients achieving BP goals increased from 55% to 72% (SBP and 60% to 71% (DBP) • No p-values were reported	High risk of bias
**Telephone based non-professional patient navigation for patients who were knowledgeable of the community resources**	Loskutova et al. 2016	USA- patients managed in primary care N = 179	Mixed methods	• Compared with baseline, reduction in HbA1c after the intervention (7.8 vs 7.2%, p = 0.001) among subgroup of patients with an existing diagnosis of T2DM	High risk of bias
**Management of patients through integrated primary/specialist model of community care (multidisciplinary clnic screening, development of patients’ specific management plan)**	Russell et al. 2013	Australia- primary care in a population with high proportion of ethnic or indigenous population N = 328	Trial	• Mean HbA1c in intervention group decreased from 70.4 mmol/mol to 60.7 mmol/mol at 12 months (mean difference -9.0; 95% CI -12.2 to -6.4, p<0.05) • After stratification into quartiles based on baseline HbA1c, the intervention group had lower HbA1c after 6 months • % participants in intervention group achieving HbA1c target of ≤53 mmol/mol (7%) increased from 21 to 42% (P<0.001); no significant increase in usual care group	Medium risk of bias
**Structured diabetes care (protocol based care, patient education program)**	Reed et al. 2001	UAE- primary care centres N = 219	Pre-post	• No statistically significant differences in baseline and post-intervention for mean fasting blood glucose (FBG), mean DBP change, mean SBP change, and total cholesterol	Medium risk of bias
**Primary care group visit model of care (patient activation, patients receiving support in group setting)**	Salinas- Martinez et al. 2009	Mexico- healthcare facility which implemented cooperative health care clinic N = 1201	Cohort	• At 15 months’ follow-up, mean FPG lower in group visit patients compared to usual care patients (155.3 ± 59.5 vs. 175.7 ± 67.7 mg/dL, p ≤0.01) • SBP and DBP lower in patients on group visits (SBP 123.6 ± 13.4 vs. 127.5 ±12.8 mmHg, p <0.01 and DBP 73.5 ± 8.5 and 79.4 ± 6.3 mmHg, p <0.01)	Low risk of bias
**Coaching and education**					
**Patient participation programme which included two 2-hr teaching sessions about ways to achieve control of modifiable risk factors**	Rachmani et al. 2005	Israel–patients referred to a diabetes clinic of an academic hospital N = 141	Randomised controlled trial	• Between baseline and 4-year follow-up, patient participation group had greater reductions in HbA1c (9.6 vs. 8.9), SBP (160 vs. 148), DBP (95 vs. 88), and LDL-C (148 vs. 124) compared to standard care group (p<0.05 for between-group differences) • Between baseline and 8-year follow-up, patient participation group had greater reductions in HbA1c (9.6 vs. 9.2), SBP (160 vs. 147), DBP (95 vs. 85), and LDL-C (148 vs. 128) compared to standard care group (p<0.05 for between-group differences)	High risk of bias
**Administration of education and health behaviour classes, delivered by nurse and dietitian, in a primary care setting**	Ridgeway et al. 1999	USA–general internal medicine patients receiving care in an ambulatory clinic N = 56	Randomised controlled trial	• After 6 months, intervention group had reductions in mean FBG (from 215 to 180mg/dl, p = 0.024), mean glycated hemoglobin (12.28% to 10.21%, p = 0.034), mean LDL-C (133 to 113 mg/dl, p = 0.313), and mean total cholesterol (59 to 221 mg/dl, p = 0.0129) •	High risk of bias
**Longer patient-physician interaction, health education forums and patients discuss their experiences of management**	Mshelia et al. 2007	Nigeria- Patients in a metabolic research unit or medial outpatient department N = 220	Trial	• Reduction in % patients with good fasting glycaemic control in the intervention group vs. control group (52.1% vs 48.8%, p<0.05) • Difference in % of patients who had food 2HPP glycaemic control in intervention group vs. control group (47.9% vs 35.4%, p<0.05)	High risk of bias
**Outpatient diabetes education programme delivered by certified diabetes educators (who are either registered nurses or dietitians)**	Kiblinger et al. 2007	USA- patients referred by physicians to attend outpatient diabetes programme N = 501	Pre-post	• Pre-post mean HbA1c decreased from 7.9% to 6.7% (p = 0.001) • Among patients with uncontrolled diabetes (HbA1c >7.0%), decrease in mean HbA1c between baseline and follow-up (9.1% vs. 7.1%, p<0.001) • Medication adherence increased from 5% to 21% for four classes of medication: antihypertensive agents, aspirin, injectable insulin, and insulin sensitizers (p = 0.001).	Medium risk of bias
**Diabetes service which provides counselling and monitoring for patients with type 2 diabetes**	Groeneveld et al. 2001	Netherlands- patients in general practice N = 246	Randomised controlled trial	• Among those with poor initial FBG (FBG >10mmol/l), mean HbA1c of intervention group patients was lower than that among control group patients (p = 0.001).	Medium risk of bias
**Structured group education program delivered in community by healthcare professionals**	Davies et al. 2008	UK- patients managed in primary care N = 824	Randomised controlled trial	• No significant mean change in HbA1c from baseline to 12 months • Reduction in triglyceride levels at 8 months: Intervention -0.57mmol/l (-0.71–-0.42), control -0.34 mmol/l (-0.53–-0.15), p = 0.008	Medium risk of bias
**Motivational interviewing and health coaching over the phone**	Browning et al. 2016	China- patients in government run community health stations N = 668	Cluster randomised pragmatic trial	• No differential treatment effect for HbA1c, with treatment and control (i.e. usual care) groups both showing improvement	Low risk of bias
**Individually tailored education by visiting nurses, assessing patients educational background and level of understanding alongside family and environmental factors**	Ko et al. 2011	South Korea–low-income patients with diabetes in a public health centre N = 96	Pre-post	• Significant relationship between the provision of individually tailored education programmes for diabetes management and FBG levels (chisq 40.11, p = 0.005)	Medium risk of bias
**Adherence information provided to physician; motivational interviewing delivered by trained staff**	Pladevall et al. 2015	USA- patients in a health system N = 1692	Randomised controlled trial	• No significant differences between groups' HbA1c and LDL-C levels at 18 months post-randomisation compared to usual care • No significant differences between groups at other time points post-randomisation (6 months, 12 months) for HbA1c, LDL-C, oral diabetes medication adherence, lipid lowering medication adherence	Medium risk of bias
**Healthcare type/setting**					
**Differences in treatment between public health centre and private clinic**	Panarotto et al. 2009	Brazil- patients managed in public health service or private clinic N = 357	Cohort	• Patients in public health centre had worse HbA1c (baseline 9.7 vs. final 8.3) data than patients in private clinic (baseline 8.3 vs. final 7.5) (baseline vs. final p<0.05, between-group difference p<0.01) • Patients in public health centre had worse cholesterol (baseline 205.1 vs. final 188.7) outcomes than patients in private clinic (baseline vs. final 205.8 vs. final 172.1) (baseline vs. final p<0.05, between-group difference p<0.01) • Frequency of visits was a determinant of better control (B = 0.72 95%CI: 0.55,-0.93, p<0.01)	Low risk of bias
**Status of diabetes control comparing primary healthcare setting vs. secondary/tertiary healthcare setting**	Tai et al. 2006	Taiwan- Primary and secondary/tertiary healthcare facilities N = 1302	Cross-Sectional	• Primary care patients had worse HbA1c data than secondary/tertiary care patients (primary care 8.4 ± 1.8% vs. secondary/tertiary care 8.1 ± 1.6%, p < 0.001) • Primary care patients had better BP control than secondary/tertiary care patients (BP target 140/90 mmHg, primary care 42.3% vs. secondary/tertiary 46.6%, p < 0.05)	Medium risk of bias
**Access and Use**				•	
**Access (i.e. insurance status, perceived trouble accessing care, perceived access to medication, and usual place of care) to healthcare on glycemic control**	Rhee et al. 2005	USA- outpatient diabetes programme catered to a largely 41-African-american population with limited financial resources and at high risk of complications N = 605	Cross-Sectional	• Health insurance was not significantly associated with HbA1c Average HbA1c levels were higher in people who reported trouble accessing medical care (9.4%, p<0.001) and in those with no prior need for care (10%, p<0.001), compared to those with no trouble getting care (8.7%) • Compared to those reporting no trouble getting medications (8.9%), those with no prior need for medications had higher HbA1c (10%, p<0.001) • Compared to those with doctor's office as usual place of care (8.6%), those seeking care at an acute facility 9.5%, p<0.001) and nowhere 10.3%, p<0.001) had higher HbA1c • Having trouble getting care was associated with a 0.57% increase in HbA1c (p = 0.04), use of an acute care facility was associated with 0.49% higher HbA1c (p = 0.047) and having gone nowhere for care was associated with 1.08% higher HbA1c compared to going to a doctor's office	High risk of bias

#### Innovative/integrated models of care

14 studies examined the impact of innovative/integrated care delivery models on outcomes. In this review, we define innovative/integrated care delivery models as multifaceted care models that bring together different components of services towards improving outcomes.

Five studies took place in Europe: one each in Spain [84], the Netherlands [73], Denmark [47], the United Kingdom [79], and Italy [97]. Three studies were set in Asia: two in China [25, 43] and one in Thailand [86]. Three studies took place in the United States [87, 104, 116], and one each was in Australia [51], the Middle East [93], and Central America [83].

Despite variation in types of innovation/integration of care employed, implementation sites, and country settings, 11 studies found positive associations between innovative/integrated models of care delivery on diabetes control and adherence outcomes. A Chinese trial among older patients with diabetes receiving hospital-based specialist care [25] found improved mean HbA1c in the group receiving integrated care compared with the traditional model. An Australian trial [51] found that mean HbA1c at 12 months significantly decreased in a group receiving integrated community care while there was no significant change in a control group.

Three cohort studies found improved outcomes. A Dutch study [73] found that mean HbA1c and the proportion of patients with poor control both fell significantly in a group receiving structured care (i.e. the implementation and practice of care processes as per clinical practice guidelines) from general practitioners but rose in the control group during 2 years of follow-up. Good control (HbA1c <7%) was achieved in 54.3% of those receiving structured care compared to 44.1% of controls (p = 0.013). A Mexican study of group management found that mean fasting plasma glucose and BP were significantly lower at 15 months’ follow-up than in controls receiving usual treatment [83]. A Spanish evaluation of a multifactorial intervention in primary care centres [84] found highly significant improvements in a wide range of biochemical parameters after one year.

A Thai case-control study [86] found significantly lower mean HbA1c and a significantly higher proportion of patients with HbA1c <7% in those managed in a community clinic emphasising promoting continuity of care compared to those receiving standard care in an outpatient hospital setting. A cross-sectional study in the United Kingdom found that an integrated "care package" [79] was associated with lower HbA1c values and increased probability of meeting cholesterol (≤4mmol/l) and BP (≤140/80mmHg) targets compared with controls receiving usual treatment.

Three other quantitative studies found positive results: a pre-post study in the United States assessing the impact of using the Asheville care management model [87], a record/chart review also in the United States looking at effects of a diabetes management programme in a university health system [104], and an Italian pre-post study assessing the impact of a structured education-based model at a diabetes clinic [97]. All three found improvements in achievement of control outcomes (e.g. proportion of patients achieving HbA1c, SBP, DBP, and cholesterol goals). A mixed-methods study in the United States evaluated a ‘Patient Navigator Model’ [116], finding lower mean HbA1c post-intervention (7.8% vs 7.2%, p = 0.01).

Two trials obtained mixed results. A Chinese trial of a case management, behaviour change-focused, protocol-driven model of care in an outpatient hospital setting [43], found that mean HbA1c was significantly reduced in the case management group at six months. A Danish trial of a structured personal care model [47] found a significant benefit only among female patients: the median HbA1c level was 8.4% in women receiving structured care vs. 9.2% in women receiving usual care. A pre-post study in the United Arab Emirates found no effect of an integrated care model on outcomes in primary care facilities [93].

#### Coaching and education

Nine studies, all quantitative, focused on the impact of delivering services including coaching and education. Four found positive effects on control and adherence.

An Israeli trial compared the impact of standard care with a participative programme including education and lifestyle modification among patients with diabetes, hypertension, and hyperlipidaemia [28]. Over a mean follow-up of 7.7 years, mean HbA1c, SBP, DBP and LDL-C values were significantly lower in the intervention group. A US trial found that nurse and dietitian-administered diabetes education and health behaviour classes delivered in primary care were associated with significant reductions in mean FBG, mean HbA1c, mean LDL-C, and mean total cholesterol [34]. A Nigerian trial examining the impact of prolonging physician-patient interaction and incorporating health education forums for patients with diabetes found a significant difference in the percentage of patients who had good fasting glycaemic control and 2-hour post-prandial glycaemic control in the intervention compared to the control group [49].

A pre-post study in the United States found that an outpatient diabetes education programme delivered by certified diabetes educators, who are either registered nurses or dietitians, was associated with decreased mean HbA1c (p = 0.001) and increased medication adherence for antihypertensive agents, aspirin, injectable insulin, and insulin sensitisers (p = 0.001). Among patients with uncontrolled diabetes (HbA1c >7.0%), there was a significant decrease in mean HbA1c between baseline and follow-up [90].

Two studies obtained mixed results. A randomised controlled trial conducted in the Netherlands found that providing counselling to patients managed in general practice had no significant effect on mean HbA1c after a year [38]. However, among those with FBG >10mmol/l, the mean HbA1c of intervention group patients was lower than that among control group patients (p = 0.001). A British randomised controlled trial examining the impact of a healthcare professional-delivered structured group education programme delivered in the community found no statistically significant differences in mean HbA1c of the structured group education arm compared to the control group [36]. However, the intervention group showed a reduction in triglyceride levels at eight months (intervention -0.57mmol/l (-0.71–-0.42) vs. control -0.34 mmol/l (-0.53–-0.15), p = 0.008).

Three studies–a Chinese randomised pragmatic trial of telephone-based motivational interviewing (MI) and health coaching [48], a South Korean pre-post study of individually-tailored diabetes education by visiting nurses on low-income patients [96], and an American randomised controlled trial of provision of adherence advice and/or MI [40] found no significant impact of coaching and education-based interventions on control and adherence.

#### Healthcare type/setting

Two studies, both quantitative, looked at the impact of healthcare type/setting on diabetes outcomes. A Brazilian cohort study compared those attending a public and private clinic [81]. While both groups showed improvements across all clinical parameters, patients receiving public healthcare had significantly higher mean HbA1c and mean cholesterol than private care patients. A Taiwanese cross-sectional study explored differences in control outcomes among patients receiving care in primary vs. secondary/tertiary healthcare settings [61] and found that patients in primary care had higher mean HbA1c values but better BP control than patients in secondary/tertiary care.

#### Access and use

A cross-sectional study in the United States examined the impact of access to healthcare on diabetes control among patients receiving care from a large two-county public health system delivering services to a vulnerable, high-risk minority ethnic group population. This study looked at different facets of access including the availability of insurance coverage, experience of seeking care, and the ability to access medication. Having trouble getting care was associated with a 0.57% increase in HbA1c (p = 0.04), use of an acute care facility was associated with 0.49% higher HbA1c (p = 0.047) and having gone nowhere for care was associated with 1.08% higher HbA1c compared to going to a doctor's office. Lack of insurance was not found to be associated with levels of HbA1c [57].

### Governance

Two included studies evaluated the effects of health system factors relating to governance challenges. Table 5 summarises the findings of studies examining associations between governance and diabetes outcomes.

**Table 5:** Summary of findings of studies examining the associations between governance and T2DM outcomes.

Health system arrangement	Study	Setting and sample size	Study design	Findings (95% CIs Given in Brackets Where Available)	Risk of Bias Assessment
**Governance**					
**Patient-doctor relationship and diagnosis of T2DM in patients**	Drivsholm et al. 2006	Denmark- newly diagnosed patients managed in general practice N = 1136	Cross-sectional	• Patients classified as not knowing their GPs well had relatively high HbA1c levels compared with levels among other patients (known well 10.2% vs. known fairly well 10.2% vs. not known well 11.3%, p<0.0001) • Patients classified as not knowing their GPs well had relatively high FBG compared with other patients (known well 13.7mmol/l vs. known fairly well 13.6 mmol/l vs. not known well 14,8mmol/l, p = 0.007)	Low risk of bias
**Trust in physicians and its moderating effect on cost-related nonadherence**	Piette et al. 2005	USA- Veteran Affairs health system N = 912	Cross-sectional	• Among patients with high levels of physician trust, rates of cost-related underuse increased (p = 0.001) 4% among patients with low monthly out-of-pocket costs (<$51) and 11% among patients with high monthly costs (>$100) • Rates of underuse increased from 4% to 30% (p = 0.01) among patients with low levels of physician trust.	High risk of bias

Two cross-sectional quantitative studies looked at the impact of patient-physician relationships on diabetes outcomes. Both studies found that the nature of patients’ relationships with physicians impacted control and/or adherence. A Danish study found that patients whom GPs classified as not knowing well had relatively higher mean HbA1c and fasting plasma glucose levels compared to those of patients classified as known “well” or “fairly well” [64]. A study in th United States found that among patients reporting high levels of physician trust, rates of cost-related medication underuse significantly increased from 4% among patients with low monthly out-of-pocket costs (<US$51) to 11% among patients with high monthly out-of-pocket costs (>US$100) [55].

### Complex interventions: Studies with more than one building block

Seven studies evaluated outcomes incorporating components from multiple health systems domains. Three took place in high-income countries [54, 72, 95], five were quantitative [54, 63, 69, 72, 95], and one was mixed-methods [115]. Three studies looked at the combined impact of more than two health systems components. Table 6 summarises the findings of studies examining associations between complex interventions and diabetes outcomes.

**Table 6:** Summary of findings of studies examining the associations between studies with more than one health systems building block and T2DM outcomes.

Health system arrangement	Study	Setting and sample size	Study design	Findings (95% CIs Given in Brackets Where Available)	Risk of Bias Assessment
**Complex interventions**					
**Studying health system change over time (reimplementation of specialist physicians for diabetes care, structured teaching and training programmes, postgraduate training courses for physicians and staff for treatment and performance of structured teaching and training programmes)**	Schiel et al. 2006	Germany- Insulin treated T2DM patients N = 323	Cohort	• Relative HbA1c improved over time (1989/90 = 9.17, 1994/5 = 9.01, 1999/2000 = 7.57: p<0.05 for 89/90 to 99/00, and 94/95 to 99/00) • % of patients with relative HbA1c <7.2% improved over time 89/90 = 23.7%, 94/5 = 18.8%, 99/00 = 3.5%: p<0.05 for 89/90 to 99/00, and 94/95 to 99/00)	Medium risk of bias
**Primary care clinics' service delivery consistency with the Chronic Care Model using ACIC scores as a measure**	Parchman et al. 2007	USA- Primary care clinics in a research network N = 618	Cross-Sectional	• The total Assessment of Chronic illness Care (ACIC) score inversely associated with HbA1C control after controlling for patient demographics and self-care behaviors, with HbA1C 0.073 points lower for each 1-point increase in ACIC score (p<0.001) • This relationship was strongest among patients who had not adhered to exercise in the past 6 months (A1C 0.1404% lower, p<0.001)	High risk of bias
**Impact of enrollment in public health insurance on blood glucose control in poor adult diabetics**	Sosa-Rubi et al. 2009	Mexico- adults with diabetes in a national survey N = 1491	Cross-Sectional	• Uninsured patients had very poor HbA1c control (>12.0%) in greater proportion than insured patients (46.2% versus 36.7%, p<0.01) • Municipalities with more health units per 1000 population had a greater likelihood of being the place of residence of those with poor HbA1c (OR: 3.17; z-statistic: 2.08) • Insured patients and people living in areas with more nurses per 1000 population had a greater likelihood of not having poor HbA1c (OR: 4.59; z-statistic: 1.75)	High risk of bias
**Performance-based provider compensation program in a disadvantaged population**	Coleman et al. 2007	USA- patients in an underserved population N = 1166	Pre-post	• Implementation of the pay for performance programme program increased the probability of receiving 2 HbA1c tests by 15.67%(p<0.0001) compared to not having the pay for performance program	Medium risk of bias
**Decentralization of diabetes services at the primary health care and community levels using a participatory local development approach**	Pilleron et al. 2014	Philippines- 14 Barangays (small administrative division; village or ward) N = 1457	Cross-sectional	• Mean HbA1c were 7.8% (SD: 1.9) and 8.5% (SD: 2.0), 62 and 69 mmol/ mol, respectively in the intervention and the control groups (p = 0.003) • % patients achieving HbA1c <6.5% (48 mmol/mol) was higher in the intervention group compared to the control group (p = 0.013)	Low risk of bias
**Different public health facility types on diabetes care (hospital with specialist(HS), hospital without specialist(HNS), health clinics with family physicians(CS) and health clinic without doctor (CND)**	Chew et al. 2013	Malaysia- patients managed in different public health facilities N = 57780	Cross-sectional	• Compared to HS (reference category), a higher proportion of CS patients achieved HbA1c≤6.5% (OR = 1.20, 95%CI = 1.06–1.37) • Compared to HS, a higher proportion of CS patients achieved BP <130/80 (OR = 1.36, 95%CI = 1.17–1.57)	Low risk of bias
**Role of Primary Healthcare nurses provided decision support, escalated scaling up of medication, and prompt access to specialist care**	Katz et al. 2009	South Africa- primary care nurses and patients in a programme modelled after the chronic care model N = 257	Mixed methods	• Programme successful in supporting Primary Health Care Nurses (PHCN)s, detecting patients with advanced disease, and ensuring early referral to a specialist center • Programme improved early detection and referral of high risk, poorly controlled patients and had an impact on PHCNs’ knowledge • Disadvantages: poor follow up due to poor existing health systems and inability to integrate into existing chronic disease services • No clinical outcomes were reported	Quantitative section High risk of bias, qualitative section low risk of bias

A South African mixed-methods study looked at the impact of service delivery, intellectual inputs, and governance, examining the effect of a programme combining the Chronic Care Model with primary care nurse support, medication scale-up, and improved access to specialist care [115]. It reported improved disease awareness through early detection and detecting patients with advanced disease, and improved treatment through referral of high-risk, poorly controlled patients. A German prospective survey found that systems-level changes, including specialist physicians, structured teaching and training programmes, postgraduate training courses for physicians and staff, and increased access to self-monitoring equipment were associated with improvement in mean HbA1c and percentage of patients with mean HbA1c <7.2% between 1989 to 2000 [72]. A Mexican cross-sectional study assessing the impacts of healthcare financing and physical and human resource inputs [58] found that uninsured patients were more likely to have mean HbA1c >12.0% than insured patients, that municipalities with more health units in relation to population had more patients with poor HbA1c (OR: 3.17), and insured patients and those living in areas with more nurses per 1,000 population had a greater likelihood of not having poor HbA1c (OR: 4.59).

Remaining studies looked at the combined impact of two health systems components. Two cross-sectional studies, one each from the United States [54] and the Philippines [69], looked at the combined impact of service delivery and intellectual inputs, with a focus on the concepts/principles of the Chronic Care Model. Both studies found positive relationships between service delivery founded in the Chronic Care Model and diabetes control (e.g. mean HbA1c) and awareness (e.g. odds of being tested for HbA1c, FBG, lipids) outcomes.

A Malaysian cross-sectional study examining the impacts of service delivery and human resource inputs on diabetes control [63] found that patients at a hospital with specialists had significantly lower mean HbA1c, LDL-C, and higher mean HDL-C than patients at a health clinic with family physicians, a health clinic without a doctor, and a hospital without specialists. Patients at a health clinic with family physician were most likely to achieve HbA1c ≤ 6.5% (aOR 1.2) and BP targets (aOR 1.4). Patients at a hospital without specialists were 3.4 times more likely to not achieve LDL-C targets. A pre-post study in the United States examining the impacts of service delivery and healthcare financing on outcomes [95] found a performance-based provider compensation programme significantly increased the probability of disadvantaged, underserved patients receiving two HbA1c tests by 15.67%.

### Health systems complexity considerations

67 of the 93 studies did not address interdependence and linkages between health system domains [25–30, 33–40, 42–46, 48–52, 56, 59, 61, 62, 65–68, 72–78, 80–87, 89–95, 97, 98, 103–107, 115, 116]. Of the studies which did, three considered linkages between human inputs and service delivery [69, 88, 99]. These studies considered the need for sufficiently trained healthcare professionals beyond simply delivery of healthcare services on outcomes.

Five studies considered interdependencies between healthcare financing and service delivery [32, 55, 100, 102, 114]. They pointed to high costs of associated services required for diabetes care and control, particularly with regard to the cost of prescription medication. One study pointed to a lack of understanding among clinicians, arguing that “physicians’ role in influencing patients’ response to medication costs [are] not well understood” [55].

Four studies considered the linkages between multiple, dynamic health system domains [108–110, 112]. These tended to study health system barriers and facilitators of diabetes care using qualitative methodologies. For example, a Tunisian study invited patients and healthcare professionals to discuss the barriers they faced in receiving and providing care. The findings were positioned in relation to several health system domains, namely inputs (i.e. human, physical, intellectual resources), service delivery, and healthcare financing [109].

14 studies considered the interactions of other factors not usually covered within the health system building blocks, but still linked to the health system. Five considered patients’ socioeconomic status [53, 57, 64, 101, 111], of which two discussed issues facing low-income patients [58, 96]. Five studies considered ethnic factors [24, 41, 60, 71, 113]. Two studies considered the role of gender [47, 70]. These studies explored how patients’ demographic characteristics might interact with different health system domains and affect outcomes.

## Discussion

This systematic review sought to assess the influence of health systems-level factors on T2DM awareness, treatment, adherence, and control. Despite the limited scope and variable quality of included articles, we identified several key health system facilitators and barriers to diabetes control, treatment, adherence, and awareness outcomes. Several studies also reported on health system factors which were neither facilitators nor barriers, showing no statistically significant impact on outcomes.

We confirmed the importance of two important health system barriers to effective diabetes care. The first was the presence of financial constraints faced by the patient. These were either self-reported or implied by the presence of co-payments for medication and were found to be a barrier to control and/or adherence outcomes. We also showed that reducing out-of-pocket payments could improve diabetes outcomes. Second, lack of access to health services and medication was a barrier to achieving good diabetes outcomes, with the evidence primarily from qualitative studies.

We also found three health system facilitators. First, integrated, innovative care models were positively associated with improved diabetes outcomes. These results are consistent with studies of integrated care programmes for other chronic diseases [117, 118]. Despite the heterogeneity of integrated care models and the national settings where they were implemented, many studies that yielded positive results had certain commonalities, namely patient education/empowerment, care continuity, data management, task-sharing, and multidisciplinary/team-based care. Yet it remains unclear what is the right mix of integrated care components and whether some are more important than others. Also, as most of these studies were in high-income countries, their applicability to low- and middle-income settings is unclear. It is also important to note the lack of consensus on definitions of “innovative” and “integrated care” [119, 120] and the components/elements that constitute an “integrated” care model [121, 122] in the health systems research community. Second, there is evidence to support pharmacist involvement. While this is consistent with findings in other chronic disease outcomes, as well as several meta-analyses [123, 124], we cannot rule out the potential of publication or reporting bias informing our findings. In this review, we surprisingly found fewer papers on nursing that we did pharmacists. This may be because nursing models are better established and therefore less novel, with fewer publications relevant to the aims of this review–but not necessarily less effective or impactful than pharmacist care models. Third, education programmes led by health professionals showed mostly positive effects on control.

Several other health systems facilitators were identified, namely peer support, positive patient-physician relationships, and multi-faceted interventions, but there is rather less evidence to draw on.

### Study strengths and limitations

This review has several strengths. It includes a wide range of measures of control, treatment, awareness, and adherence. Its conceptual framework, building on previous work by Maimaris et al [20] and Balabanova et al [13], enabled studies to be linked to different health systems domains. Our systems approach helps us map the global landscape of health systems-related research on diabetes outcomes, identifying geographical and topic gaps in research and variation in types and rigour of studies conducted, thereby informing decisions on needs for future research.

Another strength is the inclusive view of governance that encompasses the relationships and everyday practices of actors in the health system. By regarding governance as practices that are driven by macro- or meso-level decision-making, but operationalised by individuals at lower levels in the health system [125], this review harkens to the move to make health systems research a “people-centred science”, as “it is people who ultimately determine the character of a health system” in their capacities as users, providers, managers, knowledge agents, and financers [126]. It also recognises the importance of complexity in health systems research [17, 18]. This is consistent with recent calls in the health systems research literature to recognise that health systems are influenced by the settings in which they operate [126], and that health needs and outcomes are dynamic, evolving, and generated and shaped by social, economic, political, historical, and cultural forces [127].

Additionally, we did not apply language restrictions to our systematic review, which allowed us to search for, retrieve, and consider studies that were not published in English, thereby reducing language-related publication bias. We conducted searches of smaller, more regionally-focused databases (e.g. WPRIM), increasing our likelihood of capturing regional or smaller-scale research that may not have been accessible via or indexed in larger, standard academic databases.

This study is not without limitations. Heterogeneity of study designs, populations, analytical strategies, and effect measures meant it was not possible to conduct a meta-analysis. Included quantitative studies were of variable methodological quality, as evinced by the large proportion of studies rated as having an overall high risk of bias and and correspondingly small number of studies assessed to have low risk of bias across methodological domains. Although most included qualitative studies were found to have low risk of bias, it was not possible to draw causal inferences from them. As such, inferences about temporal and/or causal relationships between systems-level factors and diabetes outcomes could only be made for a limited number of factors, with careful consideration of context.

We are unable to exclude publication bias or reporting bias. For example, studies exploring effects of various factors on diabetes outcomes may have neglected to report results for health systems domains which did not achieve statistical significance. Furthermore, the review found that work from the United States was overrepresented, which provides helpful insights into gaps in the literature, but limits the applicability of our results to other contexts. Additionally, we found that limited evidence of studies assessing awareness as an outcome. Most awareness studies found in the initial search were focused on relationships between demographic and social factors (e.g. sex, age, educational level, income) on awareness, and not relationships between systems-level factors and awareness. Also, due to our focus on health systems factors and awareness, control, treatment, and adherence outcomes, it was not within this review’s scope to address broader questions around the sustainability (e.g. cost-effectiveness, cost-benefit) of interventions and programmes targeted at improving diabetes outcomes.

### Policy implications

This review found an association between minimising out-of-pocket payments (e.g. drugs, self-monitoring equipment) and improved control and adherence in the North American context. This highlights the importance of reducing, or ideally eliminating, out of pocket costs of both prescription medication and monitoring supplies. It is unfortunate that few such studies were conducted elsewhere. There seems to be potential for moving to integrated care models where these do not yet exist, taking into account synergies between the dynamic and interacting components of integrated care models, including healthcare professionals aspects of care (e.g. education, empowerment, social support, case management), approaches to care delivery (e.g. structured care, multidisciplinary team care), and information technology (e.g. clinical decision support systems, shared electronic medical records).

Previous evidence has shown that task-sharing with non-physician healthcare workers improves management of non-communicable diseases [128]. Consistent with this, our review found evidence of an association between outcomes and sharing of certain care services and processes with pharmacists. However, it is not possible to rule out the influence of publication/reporting bias, and evidence remains dominated by studies from the United States.

### Research implications

This study points to several possibilities for future research. Although we found evidence to support integrated, innovative care models, their implementation must be underpinned by a robust evidence base. As such, more studies looking at the impact of different types of care integration models on diabetes outcomes should be conducted. These studies may also generate valuable insights on which “mix” of care components and what types of integration are most conducive to achievement of positive outcomes.

There is also a need for more high-quality, longitudinal studies identifying the effect of health system arrangements on a variety of different outcomes. Studies of screening tended to be focused on uptake and adherence and not health and social outcomes. Additionally, we found diverse measures of medication adherence. There is a clear need for greater consistency here. Importantly, there were few studies focusing on upstream health system factors such as leadership and governance affecting good diabetes outcomes. For example, achieving integrated and innovated care delivery models is often dependent on effective management and lateral team management including different specialities, while expanding financial protection among larger sections of the population often results from political will; more research is needed on these associations.

Lastly, most studies are in high income countries. As the global prevalence of diabetes rises [129–131] and the influence of health systems factors on chronic disease control and management is increasingly recognised [132–134], efforts to generate evidence from low and middle-income countries (LMICs), especially considering their diverse population characteristics, diet and lifestyle shifts, sociocultural contexts, and health systems, should be augmented.

## Supporting information

S1 Table Study designs, settings, findings, and risk of bias of included studies. (DOCX)

S1 Text PRISMA 2009 checklist. (DOC)

S2 Text Medline search strategy. (DOCX)

S3 Text Risk of bias assessment tool for observational studies. (DOCX)

S4 Text Cochrane risk of bias assessment tool for randomised trials. (PDF)

S5 Text Risk of bias assessment tool for qualitative studies. (DOCX)

S6 Text Maimaris et al. 2013: The influence of health systems on hypertension awareness, treatment, and control: A systematic literature review. (PDF)
