# Supplementary material for: Assessing the influence of health systems on Type 2 Diabetes Mellitus awareness, treatment, adherence, and control: A systematic review
Source: PLoS One. 2018 Mar 29;13(3):e0195086. doi: 10.1371/journal.pone.0195086 (PMC5875848; doi:10.1371/journal.pone.0195086)
Supplement: S1 Table — (DOCX) [file pone.0195086.s001.docx]

S1 Table. Study designs, settings, findings and risk of bias of included studies.

Observational

Key

|  | High risk of bias |
| --- | --- |
|  | Unclear risk of bias |
|  | Low risk of bias |
|  | Complex intervention |

S – Selection bias

D – Differential misclassification bias

N – Non-differential misclassification bias

C – Confounding bias

Abbreviations Used

AOR – adjusted odds ratio

OR – odds ratio

SBP – systolic blood pressure

DBP – diastolic blood pressure

BP – blood pressure

T2DM – Type 2 diabetes mellitus

HMO – health maintenance organization

FFS – fee-for-service

OGTT – Oral glucose tolerance test

| **Study (Author, Year, Setting)** | **Context** | **Study Design** | **Sample Size** | **Health System framework domain** | **Health System Arrangement** | **Outcomes** | **Relevant findings (95% confidence interval given in Parenthesis)** | **Risk of Bias (Observational)** | | | |
| --- | --- | --- | --- | --- | --- | --- | --- | --- | --- | --- | --- |
|  |  |  |  |  |  |  |  | S | D | N | C |
| **Bello et al., 2012 [Nigeria] [88]** | Patients diagnosed with T2DM seeking ambulatory care at a Primary Health Facility | Pre-post | 170 | Inputs (Human resource) | Follow up intervention with pharmacists | Control | - Mean A1C reduced from 8.08 pre-intervention to 7.08 post-intervention (p<0.001) |  |  |  |  |
| **Berdine et al. 2012, [USA] [89]** | Patients in a patient-centred medical home | Pre-post | 200 | Inputs (Human resource) | Integration of pharmacist practice | Control | - Mean A1C decreased from baseline at 1 year (8.3% to 7.7%, p<0.0001) and 2 years (8.25% to 8.10%, p=0.006) |  |  |  |  |
| **Bowker et al., 2004 [Canada] [52]** | T2DM Patients managed at community pharmacies | Cross-sectional | 405 | Financing | insurance coverage for testing supplies and glycaemic control | Control | - Patients with insurance had lower HbA1c than patients without insurance (7.1 vs. 7.4, p=0.03) - Patients with insurance for testing strips had significantly lower HbA1C concentrations (adjusted difference 0.5%, p=0.006) than patients without insurance |  |  |  |  |
| **Bunting et al., 2011 [USA] [87]** | self-insured health plan members with diabetes | Pre-post | 149 | Service Delivery | web-based electronic medical record system, pharmacists, and nurses at on-site clinics, trained in use of guidelines of care; referred patients for physician follow-up | Control | - % patients achieving HbA1c goals increased from 38% to 53% - % patients achieving LDL-C goal increased from 46% to 67% - % patients achieving BP goals increased from 55% to 72% (SBP and 60% to 71% (DBP) - No p-values were reported |  |  |  |  |
| **Burge et al., 2000 [USA] [53]** | Community-wide diabetes screening programme | Cross-sectional | 118 | Financing | Socioeconomic factors on healthcare seeking behaviour of T2DM sufferers | Control | - Lack of insurance coverage as primary reason that patients with newly diagnosed diabetes fail to seek medical care (p<0.001) |  |  |  |  |
| **Chalermsri et al., 2014 [Thailand] [86]** | T2DM Patients managed at a continuity of Care Clinic run by physicians who are general internal medicine specialists | Case-control | 757 | Service Delivery | Continuity of Care(CC) Clinic compared to Outpatient Department | Control | - Mean HbA1c lower in Continuity of care (CC) clinic group vs. Outpatient department (OPD) group (7.3 vs. 7.8, p<0.001) - No. of patients who achieved HbA1c <7% in CC clinic group was 123 (32.1%) vs. 91 (24.3%) in the OPD group (p = 0.039) |  |  |  |  |
| **Chew et al., 2013 [Malaysia] [63]** | T2DM patients managed in 4 different types of public health facilities | Cross-sectional | 57780 | Service delivery + Inputs (human resources) | Different public health facility types on diabetes care (hospital with specialist(HS), hospital without specialist(HNS), health clinics with family physicians(CS) and health clinic without doctor (CND) | Control | - Compared to HS (reference category), a higher proportion of CS patients achieved HbA1c≤6.5% (OR=1.20, 95%CI=1.06 -1.37) - Compared to HS, a higher proportion of CS patients achieved BP <130/80 (OR=1.36, 95%CI=1.17-1.57) |  |  |  |  |
| **Coast-Senior et al. 1998, [USA] [94]** | T2DM patients on insulin therapy in primary care clinics | Pre-post | 23 | Inputs (Human resource) | Clinical pharmacists involved in direct patient care on the management of patients with T2DM who require insulin therapy | Control | - Decreased mean HbA1c concentrations from 11.1% to 8.9% (p=0.00004) |  |  |  |  |
| **Coleman et al., 2007 [USA] [95]** | T2DM patients in an underserved population | Pre-post | 1166 | Financing | Performance-based provider compensation program in a disadvantaged population | Awareness | - Implementation of the pay for performance programme program increased the probability of receiving 2 HbA1c tests by 15.67%(p<0.0001) compared to not having the pay for performance program |  |  |  |  |
| **de Sonnaville et al., 1997 [Netherlands] [73]** | T2DM patients managed in General practice | cohort | 359 | service delivery | Structured care at the GP, supported by allied health professionals, computerised patient register, laboratory with facilities | Control | - At 2 years, mean HbA1c decreased from 7.4 to 7.0% in structured care patients and rose from 7.4 to 7.6 % in usual care patients (p = 0.004) - % patients with HbA1c >8.5% decreased from 21.4% to 11.7% in structured care patients and rose from 23.5% to 27.9% in usual care patients (p=0.008) - HbA1c <7% achieved in 54.3% of those receiving structured care compared to 44.1% of usual are patients (p = 0.013) |  |  |  |  |
| **Drivsholm etal., 2006 [Denmark] [64]** | Newly diagnosed T2DM patients managed in General practice | Cross-sectional | 1136 | Governance | patient-doctor relationship and diagnosis of T2DM in patients | Control | - Patients classified as not knowing their GPs well had relatively high HbA1c levels compared with levels among other patients (known well 10.2% vs. known fairly well 10.2% vs. not known well 11.3%, p<0.0001) - Patients classified as not knowing their GPs well had relatively high FBG compared with other patients (known well 13.7mmol/l vs. known fairly well 13.6 mmol/l vs. not known well 14,8mmol/l, p=0.007) |  |  |  |  |
| **Elliott et al., 2013 [USA] [74]** | T2DM patients enrolled in a private health system | Cohort | 242 | Financing | Value-based insurance design, copayments for all hyperglycemic medications and supplies were eliminated | Control | - Between baseline and follow-up, no significant changes in glycemic control |  |  |  |  |
|  |  |  |  |  |  | Adherence | - Participants were more likely to self-report being adherent to oral diabetes medications at 1-year follow-up (p=0.011). |  |  |  |  |
| **Gibson, et al., 2010 [USA] [65]** | T2DM patients enrolled in a healthcare system with employer-sponsored benefits who used oral antidiabetic(OAD) with or without insulin | Cross-sectional | 152090 | Financing | Cost-sharing per prescription on OAD adherence | Adherence(PDC) | - For OAD users, the OR for non-adherence as prescription drug cost sharing increased was 0.974 (0.970- 0.984, p<0.1); for OAD-only users, the OR was 0.978 (0.973-0.984, p<0.01) - For all OAD users, the OR for non-adherence with increasing cost-sharing for physician visits was 0.996 (0.994-0.997 p<0.01); for OAD-only users the OR was 0.995 (0.993-0.996, p<0.01) |  |  |  |  |
| **Grogan et al., 2010 [USA] [66]** | Patients participating in a trial examining treatment strategies for patients with T2DM | Cross-sectional | 776 | Financing | Insurance status on T2DM control | Control | - Compared to patients with private or no insurance, patients with public insurance have lower mean A1C (private 8.2 vs. public 7.7 vs uninsured 8.29, p<0.001) and lower proportion of patients with A1C ≥7% (private 71.6% vs. public 61.2% vs. uninsured 68.3%, p=0.001) |  |  |  |  |
| **Gu et al., 2010 [USA] [75]** | Prescription drug claims data by national pharmacy benefit management company | Cohort | 12881 | Financing | Medicare Part D (Prescription Drug) Benefit coverage | Adherence(PDC) | - Patients with no coverage in the Medicare Part D coverage gap had a 38% reduction (OR=0.617, P<0.0001, 95% CI=0.523, 0.728) in odds of being adherent after reaching the Medicare Part D coverage gap, compared with patients with full coverage - Patients with only generic coverage in the Medicare Part D coverage gap had a 30% reduction (OR=0.702, P<0.001,95% CI = 0.604, 0.816) i odds of being adherent after reaching the Medicare Part D coverage gap, compared with patients with full coverage |  |  |  |  |
| **Hsu et al., 2006 [USA] [78]** | Medicare+ Choice beneficiaries enrolled in a two tier-drug plan with either a cap or no cap on annual drug costs within the Kaiser Permanente Health system | Cohort | 199179 | Financing | Limits on drug benefits for medicare beneficiaries on economic and clinical outcomes | Adherence | - For subjects with capped benefits, OR for non-adherence to antidiabetic drugs was 1.33 (1.18-1.48) |  |  |  |  |
|  |  |  |  |  |  | Control | - OR for elevated glycated hemoglobin was 1.23 (1.03-1.46) |  |  |  |  |
| **Hueston et al., 2010 [USA] [103]** | Individuals diagnosed with T2DM at a family medicine centre of a university hospital | Record review | 705 | Inputs (Human resource) | Whether having a regular physician improved quality of care for newly diagnosed T2DM sufferers | Control | - Patients with regular provider had lower average HbA1C (7.7 vs. 8.5, P=0.01) compared to patients without |  |  |  |  |
| **Hull et al., 2014 [UK] [79]** | Clinical data using diabetes registers from GPs to assess quality improvement in a primary care trust | Cohort | 41210 | Service Delivery | active patient recall for an initial appointment to collect biomedical indicies, motivational interviewing with a doctor or nurse to tailor goal setting, Financial incentives based on network achievement of targets, formation of networks of GPs, upgrading of IT | Control | - Average HbA1c value of all patients with T2DM fell from 7.80% to 7.66% between 2009 to 2012 - Achievement of cholesterol and BP targets increased from 35.3% to 46.1% |  |  |  |  |
| **Hunt et al., 2009 [USA] [80]** | Patients enrolled in a commercial exclusive provider organisation plan having 2 tiered plans with respect to cost-sharing amounts for each tier (tier 1 generic, tier 2 branded) | Cohort | 5189 | Financing | Cost sharing on OAD adherence and clinical outcomes | Control | - For each $5 increase in cost share, 0.1 increase in HbA1c (p=0.02) |  |  |  |  |
|  |  |  |  |  |  | Adherence | - For every increase in patient cost share by $1, 1.2% reduction in odds of oral diabetic medication adherence (p<0.0001) |  |  |  |  |
| **Juul et al., 2012 [Denmark] [67]** | General practices which manage T2DM patients with varying degrees of nurse-led interventions | Cross-sectional | 258 | Inputs (Human resource) | involvement of general practice nurses in T2DM care in General practice | Control | - Proportion of patients with HbA1c ≥8 was -3.7% (95%CI -6.7 to -0.6%) between practices with well-implemented nurse-led diabetes consultations compared with no nurses employed (p<0.05) |  |  |  |  |
| **Kamien et al., 1994 [Australia] [59]** | GP practitioners and patients using audit data | Cross-sectional | 204 Doctors and 467 patients | Inputs (Human resource) | Differences between vocationally (practice accreditation) and non-vocationally registered GP | Control | - No difference in HbA1c between vocationally registered and non-vocationally registered doctors |  |  |  |  |
| **Kengne et al., 2009 [Cameroon] [99]** | T2DM patients with known or newly diagnosed T2Dm not requiring insulin | Pre-post design | 225 | Inputs (Human resource) | nurses empowered to be directly in charge of running the clinics | Control | - 1.6mmol/L difference in mean FPG levels between baseline and final visit (95% CI: 0.8-2.3, p<0.001) |  |  |  |  |
| **Kiblinger et al., 2007 [USA] [90]** | Initial Visit and Follow up visit of T2DM patients referred from physicians to attend an outpatient diabetes education programme | Pre-post design | 501 | Service Delivery | Outpatient diabetes education programme delivered by certified diabetes educators (who are either registered nurses or dietitians) | Control | - Pre-post mean HbA1c decreased from 7.9% to 6.7% (p=0.001) - Among patients with uncontrolled diabetes (HbA1c >7.0%), decrease in mean HbA1c between baseline and follow-up (9.1% vs. 7.1%, p<0.001) |  |  |  |  |
|  |  |  |  |  |  | Adherence | - Medication adherence increased from 5% to 21% for four classes of medication: antihypertensive agents, aspirin, injectable insulin, and insulin sensitizers (p=0.001). |  |  |  |  |
| **Ko et al., 2011 [South Korea] [96]** | Newly registered low-income adult diabetic patients in a public health centre | Pre-post design | 96 | Service Delivery | Individually tailored education by visiting nurses, assessing patients educational background and level of understanding alongside family and environmental factors | Control | - Significant relationship between the provision of individually tailored education programmes for diabetes management and FBG levels (chisq 40.11, p=0.005) |  |  |  |  |
| **Ko et al., 2016 [USA] [100]** | T2Dm patients undergoing a medication management program in an integrated health maintenance organisation | Pre-post design | 150 | Inputs (Human resource) | Pharmacists evaluate patient therapy needs and developed a patient care plan, sessions also included teaching patients about diabates related topics, pharmacists can also adjust prescription | Control | - Intervention group had lower mean A1c readings compared with control after 1 year (8.18% vs 8.69%, p=0.012) and 2 years (8.06% vs 8.67%, p=0.014) - 2-year average decrease in A1c was larger in intervention group compared with usual care group (-1.24 vs -0.59, p=0.009) |  |  |  |  |
| **Kocarnik et al., 2012 [USA] [77]** | T2DM patients managed in primary care clinics within the VA health system | Cohort | 280603 | Inputs (Human resource) | Presence of pharmacist in clinics | Adherence | - No statistically significant effect of pharmacist presence on patients’ medication adherence |  |  |  |  |
| **Littenberg et al., 2006 [USA] [68]** | T2DM patients managed in primary care | Cross-sectional | 781 | Inputs (Physical) | Travel burden to primary care on insulin use | Adherence | - OR 0.97 (0.95-0.99) for insulin use per km of driving distance |  |  |  |  |
| **Maschak-Carey et al., 1999 [USA] [104]** | High risk patients with newly diagnosed type 1 or 2 diabetes, patients who were recently sent in the emergency department or who had been admitted to the hospital for diabetes-related problems | Record review | 1779 | Service delivery | Establishing patient education goals, hiring diabetes nurse care managers and developing clinical practice guidelines, delivering appropriate pharmacological therapies, conducting regular laboratory evaluations and specialist referral | Control | - Before enrolment, average HbA1c values were 9.03 and fell in study participants to 8.3 (p=0.03). |  |  |  |  |
| **Monte et al., 2009 [USA] [91]** | T2DM patients in a regional primary care group | Pre-post study | 50 | Inputs (Human resource) | Pharmacist-led, patient-centred pharmacotherapy management program (consultation pharmacists where patients were instructed on blood glucose monitoring and dietary practices) | Control | - At 6 months and 12 months, A1C and fasting plasma glucose (FPG) reduced compared to baseline (6 months: A1C, -1.1%, p<0.0001 and FPG, -39 mg/dL, p=0.003. 12 months: A1C -1.1% , p<0.0001and FPG -35 mg/dL, p=0.005) |  |  |  |  |
| **Musacchio et al., 2010 [Italy] [97]** | Diabetes clinic in Cusano Milanino, the Integration Operational Unit and Centre for Diabetes Care, in Milan | Pre-post design | 1004 | Service Delivery | Structured education programme based on patient empowerment where the patient is their own case manager, and managed by a multidisciplinary team | Control | - % of patients with HbA1c ≤7% increased from 32.7% to 45.8% (p < 0.0001) after 12 months follow-up - % of patients with HbA1c ≥9% decreased from 10.5% to 4.3% (p < 0.0001) after 12 months follow-up - % of patients with LDL-C < 100 mg/dl increased from 39.7% to 47.3% (p < 0.0001) after 12 months follow-up - % of patients with LDL ≥ 130 mg/dl decreased from 26.6% to 19.7% (p<0.001) after 12 months follow-up |  |  |  |  |
| **Ngo-Metzger et al., 2012 [USA] [60]** | Racially/ethnically diverse T2DM patients from various outpatient clinics | Cross-sectional | 1361 | Financing | Impact of self-reported financial pressures on cost related medication adherence | Adherence | - Perceived financial burden as associated with HbA1c ≥8% (aOR=1.7, 95%CI 1.09-2.63) - Being uninsured (aOR=1.9, 95%CI 1.13-3.21) and non-adherence (aOR=1.49, 95%CI 1.06-2.08) associated with HbA1c |  |  |  |  |
| **Oyetayo et al., 2011 [USA] [71]** | Hispanic T2DM patients seeking diabetes care in a pharmacy network | Cohort | 126 | Inputs (Human resource) | Face to face meeting with Community pharmacists every 3 months | Control | - Reductions in FPG (163 vs. 149 mg/dL, p=0.019), SBP (87 vs. 85 mmHg, p=0.003), and triglycerides (191 vs. 176 mmHg, p=0.003) - Subgroup analyses of patients with poorly controlled diabetes at baseline revealed reductions in mean HbA1c (9.2% vs 8.6%, p=0.006), SBP (147 vs. 143 mmHg, p=0.031), DBP (91 vs. 87 mmHg, p<0.001), triglycerides (259 vs. 219 mg/dL, p<0.001), LDL-C (139 vs. 123 mmHg, p<0.001), and total cholesterol (237 vs. 222 mmHg, p=0.008) |  |  |  |  |
| **Panarotto et al., 2009 [Brazil] [81]** | T2DM patients managed at either public health service or private clinic | Cohort | 357 | Service Delivery | Differences in treatment between public health centre and private clinic | Control | - Patients in public health centre had worse HbA1c (baseline 9.7 vs. final 8.3) data than patients in private clinic (baseline 8.3 vs. final 7.5) (baseline vs. final p<0.05, between-group difference p<0.01) - Patients in public health centre had worse cholesterol (baseline 205.1 vs. final 188.7) outcomes than patients in private clinic (baseline vs. final 205.8 vs. final 172.1) (baseline vs. final p<0.05, between-group difference p<0.01) - Frequency of visits was a determinant of better control (B=0.72 95%CI: 0.55,-0.93, p<0.01) |  |  |  |  |
| **Parchman et al., 2007 [USA] [54]** | 20 autonomous primary care clinics who are part of the South Texas Ambulatory Research Network | Cross-sectional | 618 | Service delivery + Inputs (intellectual) | Primary care clinics' service delivery consistency with the Chronic Care Model (measure: ACIC score) | Control | - The total Assessment of Chronic illness Care (ACIC) score inversely associated with HbA1C control after controlling for patient demographics and self-care behaviors, with HbA1C 0.073 points lower for each 1-point increase in ACIC score (p<0.001) - This relationship was strongest among patients who had not adhered to exercise in the past 6 months (A1C 0.1404% lower, p<0.001) |  |  |  |  |
| **Patel et al., 2006 [USA] [92]** | Outpatient medication assistance programme of the University of Connecticut's School of Pharmacy and Hartford Hospital | Pre-Post | 143 | Financing | Pharmacy-managed medication assistance programme for diabetic outpatients: uninsured patients receive medications free of charge | Control | - 0.85% reduction in HbA1c (0.34-1.37, p=0.002) - 33% increase in patients who achieved HbA1c <7% (p=0.008) - Total cholesterol decreased by 25.7mg/dl (11.1-40.2. p=0.001) |  |  |  |  |
| **Pawaskar et al., 2010 [USA] [82]** | State-level Medicaid patients from across 8 different states of the US, including at least 1 state from each US region | Cohort | 8581 | Financing | Capitation vs. fee for service | Adherence (MPR) | - Patients in capitated plans had 5% lower mean oral antidiabetic medication adherence than those in fee for service plans (p<0.05) - Odds ratio of adherence among patients in capitated health plans was 0.89 (0.82-0.98, p=0.05) of medication adherence compared to patients in fee for service plans |  |  |  |  |
| **Piette et al., 2005 [USA] [55]** | 5 Department of Veterans Affairs health systems | Cross-sectional | 912 | Governance | Trust in physicians and its moderating effect on cost-related nonadherence | Adherence | - Among patients with high levels of physician trust, rates of cost-related underuse increased (p=0.001) 4% among patients with low monthly out-of-pocket costs (<$51) and 11% among patients with high monthly costs (>$100) - Rates of underuse increased from 4% to 30% (p=0.01) among patients with low levels of physician trust. |  |  |  |  |
| **Piette et al., 2004 [USA] [56]** | 3 Veterans Affairs health systems in California and Wisconsin, a county healthcare system in San Francisco, and a university-based healthcare system in San Francisco | Cross-sectional | 766 | Financing | Health insurance status and its effect on cost-related medication underuse | Adherence | - Patients with private insurance almost twice as likely to report underusing medication in the prior 12 months as VA patients (P <0.0001) - Patients who reported cost-related medication underuse had an average HbA1c of 8.7% (p<0.001) - Cost-related medication underuse associated with 0.6% (0.2-0.9) absolute increase in HbA1c levels (p=0.005) |  |  |  |  |
| **Pilleron et al., 2014 [Philippines] [69]** | 14 barangays in Davao City, Mindanao, Philippines | Cross-sectional | 1457, of which n=1078 intervention and n=379 control | Service delivery + Inputs (intellectual) | Decentralization of diabetes services at the primary health care and community levels using a participatory local development approach, founded on the Chronic Care Model (CCM) | Control | - Mean HbA1c were 7.8% (SD: 1.9) and 8.5% (SD: 2.0), 62 and 69 mmol/ mol, respectively in the intervention and the control groups (p=0.003) - % patients achieving HbA1c <6.5% (48 mmol/mol) was higher in the intervention group compared to the control group ( p = 0.013) |  |  |  |  |
| **Pinsky et al., 2011 [USA] [105]** | United States, nationwide | Record review | 8011 | Inputs (Human resource) | Certification of physicians that provide high quality diabetes care | Adherence | - Patients managed by certified physicians (certification recognises physicians and practices providing high-quality diabetes care) more likely to receive prescriptions for oral antihyperglycemic agents than those managed by non-certified physicians (mean prescriptions per patient per month 0.49 vs. 0.46, p=0.02) |  |  |  |  |
| **Quinones et al., 2016 [USA] [102]** | Patients who were discharged from clinical pharmacist services in a large urban healthcare system (Parkland Health and Hospital System) in Dallas, Texas | Chart review | 915 | Inputs (Human resource) | Patients seen by clinical pharmacy specialists for diabetes management: approx 5.3 face to face visits with CPS per patient | Control | - Average HbA1c difference of -2.6% from initial to final visit (2010-2013, all p<0.01) - Increase in patients with DM achieving HbA1c >7% 2.1% in 2010, 2.7% in 2011 and 2012, and 3.1% in 2014 (all p<0.01) |  |  |  |  |
|  |  |  |  |  |  | Adherence | - Overall increases in absolute proportion of medication adherence at time of discharge: 42.8% in 2010, 43.5% in 2011, 42.8% in 2012, 49% in 2013 |  |  |  |  |
| **Reed et al., 2001 [UAE] [93]** | Three urban primary care centres in the Al Ain federal health district, United Arab Emirates, with six primary care centres used as controls | Pre-post | n=219 of which n=109 intervention and n=110 control | Service delivery | Structured diabetic care for primary care clinics | Control | - No statistically significant differences in baseline and post-intervention for mean fasting blood glucose (FBG), mean DBP change, mean SBP change, and total cholesterol |  |  |  |  |
| **Rhee et al., 2005 [USA] [57]** | An outpatient diabetes programme affiliated with a large two-county public health system with a hospital and several community primary care site, which delivers care to a predominantly African-American population with limited financial resources but at high risk for diabetes-related complications | Cross-sectional | 605 | Service Delivery | The impact of access to healthcare on glycemic control | Control | - Health insurance was not significantly associated with HbA1c Average HbA1c levels were higher in people who reported trouble accessing medical care (9.4%, p<0.001) and in those with no prior need for care (10%, p<0.001), compared to those with no trouble getting care (8.7%) - Compared to those reporting no trouble getting medications (8.9%), those with no prior need for medications had higher HbA1c (10%, p<0.001) - Compared to those with doctor's office as usual place of care (8.6%), those seeking care at an acute facility 9.5%, p<0.001) and nowhere 10.3%, p<0.001) had higher HbA1c - Having trouble getting care was associated with a 0.57% increase in HbA1c (p=0.04), use of an acute care facility was associated with 0.49% higher HbA1c (p=0.047) and having gone nowhere for care was associated with 1.08% higher HbA1c compared to going to a doctor's office |  |  |  |  |
| **Richardson et al., 2014 [USA] [106]** | Two ambulatory care internal medicine modules serving 3677 adult T2DM patients in Kaiser Permanente Georgia (KPGA) | Chart review | 28 | Inputs (Human resource) | Nurse practitioners in collaborative practices with primary care clinicians in helping improve control of HbA1c, BP, and LDL-C in adults with uncontrolled hyperglycemia | Control | - Post-intervention, 13 patients (50%) achieved HbA1c <8% compared to pre-intervention (0 patients) (p<0.001) |  |  |  |  |
| **Salinas-Martinez et al., 2009 [Mexico] [83]** | Region in northeastern Mexico that has implemented the Cooperative Health Care Clinic model | Cohort | 1201 of which n=600 group visit and n=601 usual care | Service Delivery | Primary care group visit model of care, with emphasis on patient activation, patients supporting one another in a group setting | Control | - At 15 months’ follow-up, mean FPG lower in group visit patients compared to usual care patients (155.3 ± 59.5 vs. 175.7 ± 67.7 mg/dL, p ≤0.01) - SBP and DBP lower in patients on group visits (SBP 123.6 ± 13.4 vs. 127.5 ±12.8 mmHg, p <0.01 and DBP 73.5 ± 8.5 and 79.4 ± 6.3 mmHg, p <0.01) |  |  |  |  |
| **Schiel et al., 2006 [Germany] [72]** | Insulin-treated Type 2 diabetics aged 16 to 60 living in Jena, Thuringia, Germany | Cohort | 323, of which 1989/90 = 59, 1994/5 = 117, 1999/2000 = 147 | Service delivery + inputs (human resources) | Health system changes over time, Reimplementation of specialist physicians for diabetes care, structured teaching and training programmes, postgraduate training courses for physicians and staff for treatment and performance of structured teaching and training programmes | Control | - Relative HbA1c improved over time (1989/90 = 9.17, 1994/5= 9.01, 1999/2000 = 7.57: p<0.05 for 89/90 to 99/00, and 94/95 to 99/00) - % of patients with relative HbA1c <7.2% improved over time 89/90 = 23.7%, 94/5= 18.8%, 99/00=3.5%: p<0.05 for 89/90 to 99/00, and 94/95 to 99/00) |  |  |  |  |
| **Schmittdiel et al., 2009 [USA] [70]** | Kaiser Permanente North California, an integrated healthcare delivery system providing healthcare services to 3.2 million members in Northern California | Cross-sectional | 157458 | Inputs (Human resource) | Patient gender, physician gender, and gender concordance with CVD risk factor levels (HbA1c, LDL-C, SBP) and medication adherence | Control | - Female patients of female physicians most likely to have HbA1c<8% (70% vs. 66%–68%) - Patient and physician gender interaction associated with significant LDL-C control, with male patients of male physicians having the highest proportion of patients at or below target |  |  |  |  |
| **Sosa-Rubi et al., 2009 [Mexico] [58]** | Poor adults with diabetes in Mexico | Cross-sectional | 1491 | Financing + inputs (physical + human resource) | Impact of enrollment in public health insurance on blood glucose control in poor adult diabetics | Control | - Uninsured patients had very poor HbA1c control (>7.0%) in greater proportion than insured patients (46.2% versus 36.7%, p<0.01) - Municipalities with more health units per 1000 population had a greater likelihood of being the place of residence of those with poor HbA1c (OR: 3.17; z‑statistic: 2.08) - Insured patients and people living in areas with more nurses per 1000 population had a greater likelihood of not having poor HbA1c (OR: 4.59; z-statistic: 1.75) |  |  |  |  |
| **Soumerai et al., 2004 [USA] [107]** | Diabetic patients of Harvard Vanguard Medical Associates, multispeciality group serving 300,000 people in and around Boston, MA, who are insured by Harvard Pilgrim Health Care (HPHC), a large HMO in New England | Time-series design | 3219 | Financing | Impact of policy providing free blood glucose monitors on self-monitoring of blood glucose, regularity of medication use, and improved glycemic control | Control | - Initiation of self-monitoring (as a result of financial coverage) not associated with improved HbA1c levels in those with good or adequate baseline glycemic control - Among those with poor glycemic control, initiators of self-monitoring lowered their mean HbA1c level by 0.63% compared with noninitiators (1.14 - 0.12, p=0.03) |  |  |  |  |
|  |  |  |  |  |  | Adherence | - Compared with noninitiators of self-monitoring, initiators had improvements in regularity of medication use by 6 months after initiation: −19.5 days between dispensings among those with low refill regularity (27.7−11.3); −9.7 days among those with moderate regularity (12.3−7.1), and mean HbA1c level reduced by 0.63% (1.14% -0.12%) - Among those with moderate refill regularity by 6 months after initiation of self-monitoring, initiators reduced mean gaps between dispensings by 9.7 days compared with noninitiators (12.3−7.1) - Among those with low baseline refill regularity, initiators of self-monitoring had immediate reductions in mean gaps of 19.5 days compared with noninitiators (27.7−11.3) |  |  |  |  |
| **Spence et al., 2014 [USA] [76]** | Kaiser Permanente Southern California, an integrated healthcare system providing services to 3.6 million members living in Southern California | Cohort | 2957, of which intervention n=1480, usual care n=1477 | Inputs (Human resource) | Outpatient Clinical Pharmacist Programme | Control | - Mean HbA1c in intervention group lower than usual care group (8.48 vs. 8.80, P = 0.024) - Reduction in HbA1c from baseline (-1.25 vs. -0.75, P= 0.001) |  |  |  |  |
|  |  |  |  |  |  | Adherence | - 53.5% of intervention group adherent to diabetes medications after 1 year, compared with 37.4% in the usual care group (P = 0.001) - Intervention group saw increase in medication possession ratio (MPR) from baseline compared with usual care group (0.19 vs. 0.15, P=0.024 - Intervention patients less likely to discontinue diabetes medications (11.7% vs. 35.5%, P < 0.001) and more likely to have their medication prescription filled within 30 days after the end of their supply of the last prescription post-first consultation date (34.8% vs. 12.9%, P< 0.001) - Average days to first medication prescription filled after first consultation date was 79.3 for the intervention group compared with 156.3 for the usual care group (P< 0.001) |  |  |  |  |
| **Spigt et al, 2009 [Netherlands] [101]** | Ten primary care centres comprising 44 GPs, located in the Netherlands and linked to the Maastricht University Registration Network | Record review | 58,919 and 2582 T2DM patients | Inputs (Human resource) | GP screening activity and its impact on diagnosed diabetes prevalence | Awareness | - Diabetes patients in primary care had worse HbA1c than patients in secondary/tertiary care (pri care 8.4 ± 1.8% vs. sec/ter care 8.1 ± 1.6%, p < 0.001) - Diabetes patients treated in primary care setting had better BP control than those in secondary/tertiary setting (BP target 140/90 mmHg, pri care 42.3% vs. sec/ter 46.6%, p < 0.05) |  |  |  |  |
| **Tai, 2006 [Taiwan] [61]** | Primary healthcare stations and secondary/tertiary healthcare facilities across Taiwan | Cross-sectional | 1302 | Service Delivery | Status of diabetes control comparing primary healthcare setting vs. secondary/tertiary healthcare setting | Control | - Primary care patients had worse HbA1c data than secondary/tertiary care patients (primary care 8.4 ± 1.8% vs. secondary/tertiary care 8.1 ± 1.6%, p < 0.001) - Primary care patients had better BP control than secondary/tertiary care patients (BP target 140/90 mmHg, primary care 42.3% vs. secondary/tertiary 46.6%, p < 0.05) |  |  |  |  |
| **Tan, 2015 [USA] [62]** | United States, nationwide | Cross-sectional | Commercial n = 197941, Medicare n=184439, Medicaid n=70003 | Financing | Adherence (PDC, proportion of days covered) based on insurance status: commercial, Medicare, Medicaid | Control | - Diabetes control was highest at 68.9% for commercially insured patients (69.1-68.7) than 53.7% for Medicare (53.5-54.0) and 52.7% for Medicaid patients (52.3-53.0) (p<0.05) |  |  |  |  |
|  |  |  |  |  |  | Adherence | - Average PDC and drug adherence were higher at 83% (82.9-83.1) for patients insured by Medicare than 76.6% (76.5-76.8) for patients who were commercially insured and 74.4% for Medicaid insured (74.2-74.6) (p<0.05) |  |  |  |  |
| **Tranche, 2005 [Spain] [84]** | Primary care centres in Spain | Cohort | 3466 | Service Delivery | A simultaneous, multifactorial intervention involving nutritional-hygienic measures, smoking cessation, and intensification of pharmacologic treatment carried out by primary care physicians following clinical practice guidelines | Control | - Significant results (p<0.001) for baseline vs end point % patients achieving HbA1c target <7.5% (74.9% vs 90.6%), all BP goals (<130/85: 3.5% vs 23.3%, <130/80: 1.8% vs 13.6%, <140/90: 15.2% vs 72.4%), and lipid goals (LDL <130 and HDL >40mg/dl: 5.9% vs 40.9%, triglycerides <200mg/dl: 75.2% vs 89.8%) - Significant results (p<0.001) for all indicators comparing baseline and final visit measurements: SBP (149.7 vs 133), DBP (88.6 vs 79.5), total cholesterol (223.4 vs 202), LDL-C (142.1 vs 124.1), HDL-C (49.9 vs 52.7), triglycerides (158.7 vs 139.4), HbA1c (6.9 vs 6.5) |  |  |  |  |
| **Vella et al., 2013 [Malta] [98]** | Pharmacies in Malta under the Pharmacy of Your Choice scheme, run by the Maltese government | Pre-post design | 30 | Inputs (Human resource) | Pharmacist intervention to improve glycemic control and medication adherence | Adherence | - Improvement from 24 patients reporting rarely missing a dose of medication pre-intervention to 8 patients post-intervention - Pre-intervention 1 patient reported to "never miss a dose" of medication"; increased to 22 post-intervention |  |  |  |  |
| **Ziemer et al., 2005 [USA] [85]** | Medical Clinic and Diabetes Clinic of Grady Health System, Atlanta, GA | Cohort | 438 | Inputs (Human resource) | Clinical inertia: inadequate intensification of therapy by the provider and its impact on outcomes | Control | - Tendency of individual providers to intensify therapy associated with lower HbA1C levels (P < 0.0001) - 10% higher frequency of intensification associated with a 0.15% lower level of A1C - A single episode of intensification of therapy associated with an average 0.7% A1C reduction |  |  |  |  |

RANDOMISED TRIALS

Key

|  | High risk of bias |
| --- | --- |
|  | Unclear risk of bias |
|  | Low risk of bias |
|  | Complex intervention |

S – Selection

P – Performance

D – Detection

A – Attrition

R - Reporting

| **Study (Author, Year, Setting)** | **Context** | **Study Design** | **Sample**  **Size** | **Health System framework domain** | **Health System Arrangement** | **Outcomes** | **Relevant findings (95% confidence interval given in Parenthesis)** | **Risk of Bias (Randomised Trials)** | | | | |
| --- | --- | --- | --- | --- | --- | --- | --- | --- | --- | --- | --- | --- |
|  |  |  |  |  |  |  |  | S | P | D | A | R |
| **Babamoto et al., 2009 [USA] [24]** | Hispanic/Latino T2DM patients managed in 3 inner-city family health centres | Randomised controlled trial | 318 | Inputs (Human resource) | Community health workers compared to usual clinic diabetes care (Standard provider and case management) | Control | - Mean A1C decreased from 8.6% to 7.2% (p<0.05) in the community health worker group, 8.5% to 7.4% (p<0.05) in the case management group, 9.5% to 7.4% (p<0.05) in the standard provider care group |  |  |  |  |  |
|  |  |  |  |  |  | Adherence | - Proportion of patients who reported never forgetting to take their medication decreased in case management (77%to 55%, p <0.05) and standard provider care groups (67% to 50%, p < 0.05) - Proportion of patients reporting never forgetting to take their medication decreased in case management (77%to 55%, p <0.05) and standard provider care groups (67% to 50%, p <0.05) |  |  |  |  |  |
| **Browning et al., 2016 [China] [44]** | T2DM sufferers receiving care at government run community Health stations | Cluster randomised pragmatic trial | 668 | Service Delivery | Motivational interviewing and health coaching over the phone | Control | - No differential treatment effect for HbA1c, with treatment and control (i.e. usual care) groups both showing improvement |  |  |  |  |  |
| **Chao et al., 2015 [China] [25]** | Diabetic patients receiving care from an endocrinology clinic at a Nanjing district hospital | Randomised controlled trial | 100 | Service Delivery | Integrated health management model (health record establishment, health evaluation and health management) | Control | - Mean FBG in the management group -0.82 mmol/l vs. usual care group +0.06m/mol (p=0.042) |  |  |  |  |  |
| **Cohen et al., 2011, [USA] [26]** | T2DM patients at a Veteran's affairs medical centre | Randomised controlled trial | 99 | Inputs (Human resource) | Pharmacist-led shared medical appointments program | Control | - Compared to standard primary care, treatment group had significant reductions in A1C (-0.41; 95% CI-0.74 to -0.07%, p<0.05). - Compared to standard primary care, treatment group had higher adjusted odds of achieving A1C goals (aOR, 2.73; 95% CI 1.03 to 7.16, p<0.05) and SBP goals (adjusted odds ratio, 3.06; 95% CI, 1.31 to 7.16, p<0.05) |  |  |  |  |  |
| **Davies et al., 2008, [UK] [36]** | T2DM patients managed in primary care | Randomised controlled trial | 824 | Service Delivery | Structured group education programme delivered in the community by healthcare professionals | Control | - No significant mean change in HbA1c from baseline to 12 months - Reduction in triglyceride levels at 8 months: Intervention -0.57mmol/l (-0.71 – -0.42), control -0.34 mmol/l (-0.53 – -0.15), p=0.008 |  |  |  |  |  |
| **Edelman, 2015, [USA] [37]** | T2DM patients enrolled in primary care, who have both diabetes and Hypertension which both are poorly controlled | Randomised controlled trial | 377 | Inputs (Human resource) | Nurse behavioural management of Diabetes and Hypertension in community practices | control | - No A1c and SBP differences between intervention group compared to control group, who received calls which were not tailored and discussed topics not relevant to diabetes or hypertension management |  |  |  |  |  |
| **Groeneveld et al., 2001, [Netherlands] [38]** | T2DM patients managed in General practice | Randomised controlled trial | 246 | Service Delivery | Diabetes service which provides counselling and monitoring for type 2 diabetes patients | Control | - Among those with poor initial FBG (FBG >10mmol/l), mean HbA1c of intervention group patients was lower than that among control group patients (p=0.001). |  |  |  |  |  |
| **Heisler M, 2012, [USA] [27]** | Patients with T2DM and poor blood pressure control and poor refill adherence or insufficient medication intensification managed in outpatient primary care clinics | cluster randomised pragmatic trial | 4622 | Inputs (Human resource) | Pharmacists trained in motivational interviewing on patient-centred approaches to achieving health goals, working with patients on intervention teams in person or over the phone | Control | - Mean SBPs of intervention group decreased to a larger extent as compared to the control group, who received usual care, after intervention (difference of -2.4mmHg, 95%CI -3.4 - -1.5, p<0.001) |  |  |  |  |  |
| **Heisler et al., 2010, [USA] [45]** | Veterans who have poor glycemic control currently managed under nurse care management | Randomised controlled trial | 244 | Inputs (Human resource) | Reciprocal peer support(RPS) programme conducted by other diabetic patients in group sessions and one-to-one telephone conversations | Control | - Mean HbA1c levels in intervention group reduced (-0.29%); mean HbA1c levels control (nurse case management) group increased (0.29%) (between-group difference 0.58%, p=0.004). - Among patients with baseline HbA1c >8.0%, intervention group had a mean decrease of 0.88%, compared with a 0.07% decrease in control group (between-group difference 0.81%, p<0.001) |  |  |  |  |  |
| **Houweling et al., 2009 [Netherlands] [29]** | T2DM patients referred from general practitioners to diabetes outpatient clinics of hospitals | Randomised controlled trial | 93 | Inputs (Human resource) | Transferring well-defined routine aspects in diabetes care to supervised nurses specialised in diabetes within the secondary care setting inclusing prescription of medication | Control | - After 1 year, more intervention group patients (2.2% to 33.3%, p<0.002) achieved target HbA1c <7% compared to control group who received standard care (10. 5% to 26.3%) |  |  |  |  |  |
| **Houweling et al., 2011 [Netherlands] [28]** | Patients with T2DM managed in general practice | Randomised controlled trial | 230 | Inputs (Human resource) | Transferring diabetes management to practice nurses in primary care setting | Control | - No significant between-group differences with respect to reduction in HbA1c, blood pressure and lipid profile - Control group received conventional care from general practitioners |  |  |  |  |  |
| **Jacobs et al., 2012 [USA] [30]** | T2DM patients with uncontrolled HbA1c managed in primary care setting | Randomised controlled trial | 2121 | Inputs (Human resource) | pharmacist-patient clinic visits including medication review, performing targeted physical assessment, educating on diabetes pathophysiology and importance of control; reviewing and modifying patients medication therapy, providing reinforcement of dietary guidelines and exercise | Control | - Greater absolute % change in A1c from baseline for intervention group than control group who received usual care directed by their physicians (-0.18 vs -0.8%) (p<0.05) |  |  |  |  |  |
| **Johnson et al., 2006, [Canada] [39]** | Patients with T2DM but without private insurance and not using insulin | Randomised controlled trial | 458 | Financing | Free testing supplies for self-monitoring of blood glucose Vs. patients paying "out-of pocket" for testing supplies | Control | - Reducing financial barriers by providing free testing strips did not significantly improve glycaemic control in patients |  |  |  |  |  |
| **King et al., 2009 [USA] [31]** | T2DM patients managed in primary care | Randomised controlled trial | 135 | Inputs (Human resource) | Clinic nurse practitioners had visits to a diabetes specialist, conference calls, guidance by faxing flow sheets to specialist and 24-hour reponse to critique treatment plan | Control | - No significant reduction in HbA1c from baseline comparing treatment vs. control group - The control group was not contacted, and only their charts were reviewed by the research team. |  |  |  |  |  |
| **Krass et al., 2007, [Australia] [32]** | T2DM patients collecting medication at community pharmacies | Randomised controlled trial | 289 | Inputs (Human resource) | Pharmacist-delivered, community pharmacy-based diabetes care and management programme, including support for blood glucose self-monitoring, education, adherence support, and reminders of checks for complications | Control | - Mean reduction in HbA1c in the intervention group was -0.97%( -0.8, -1.14) compared with -0.27% (-0.15,-0.39) in the control group who received usual care from pharmacists. |  |  |  |  |  |
| **Manders et al., 2016 [Netherlands] [49]** | T2DM or type 1 patients admitted to a medical centre and part of a Nurse Driven Diabetes In-Hospital Treatment protocol | Trial design | 410 | Inputs (Human resource) | Intervention included, involvement of all non-ICU nurses; specific training and obligatory certification; and independent authority of all certified nurses to initiate the correctional insulin algorithm in patients with diabetes | Control | - No significant differences in mean blood glucose and FPG levels between intervention and control groups - Control group was derived from patients hospitalised prior to implementation of the intervention. |  |  |  |  |  |
| **McDermott et al., 2015 [Australia] [41]** | Poorly controlled T2DM patients in remote indigenous communities managed in primary care | Randomised controlled trial | 213 | Inputs (Human resource) | Training indigenous health worker resident in the community to work as part of the primary care team. | Control | - At 18 months follow-up, HbA1c reduction in the intervention group (10.8% to 9.8%) was greater than reduction in control group (10.6% to 10.3%), p=0.0018 - The control group was placed on an intervention waitlist, and given the intervention after the study. |  |  |  |  |  |
| **Mshelia DS, 2017 [Nigeria] [48]** | T2DM patients from a metabolic research unit or medical outpatient department | Trial design | 220 | Service Delivery | longer patient-physician interaction, health education forums and patients discuss their experiences of management | Control | - Reduction in % patients with good fasting glycaemic control in the intervention group vs. control group (52.1% vs 48.8%, p<0.05) - Difference in % of patients who had food 2HPP glycaemic control in intervention group vs. control group (47.9% vs 35.4%, p<0.05) |  |  |  |  |  |
| **Nielsen et al., 2006 [Denmark] [47]** | T2DM patients receiving care in primary care settings | Cluster randomised pragmatic trial | 874 | Service Delivery | Structured personal care, which includes quarterly consultations and individualized goal setting for important risk factors | Control | - Median HbA1c level was 8.4% in women receiving structured care vs. 9.2% in women receiving usual care (p<0.001) - Women receiving usual care had HbA1c levels 1.1 times higher than women receiving structured care (1.06-1.14, p<0.001). |  |  |  |  |  |
| **Odegard et al., 2005 [USA] [33]** | Poorly controlled T2DM patients managed in primary care | Randomised controlled trial | 77 | Inputs (Human resource) | Meeting with pharmacist to initiate diabetes care plan followed by weekly visits or telephone calls to facilitate diabetes management | Control | - Mean HbA1c in both intervention and usual care groups decreased from baseline 6 and 12 months (p=0.001), but intervention groups achieved HbA1c decreases with fewer physician visits |  |  |  |  |  |
| **Pladevall et al., 2015 [USA] [40]** | Adult members of a health system in southeast Michigan and metropolitan Detroit | Randomised controlled trial | 1692 | Service delivery | Adherence information provided to physician; motivational interviewing delivered by trained staff | Adherence | - No significant differences between groups' HbA1c and LDL-C levels at 18 months post-randomisation compared to usual care - No significant differences between groups at other time points post-randomisation (6 months, 12 months) for HbA1c, LDL-C, oral diabetes medication adherence, lipid lowering medication adherence |  |  |  |  |  |
| **Rachmani et al., 2005 [Israel] [116]** | Patients with T2DM, hypertension, and hyperlipidemia referred for consultation to diabetes clinic of an academic hospital in Tel Aviv from 1995 to 1996 | Randomised controlled trial | 141 | Service Delivery | Patients either received standard care or participated in a patient participation programme which included two 2-hr teaching sessions about ways to achieve control of modifiable risk factors | Control | - Between baseline and 4-year follow-up, patient participation group had greater reductions in HbA1c (9.6 vs. 8.9), SBP (160 vs. 148), DBP (95 vs. 88), and LDL-C (148 vs. 124) compared to standard care group (p<0.05 for between-group differences) - Between baseline and 8-year follow-up, patient participation group had greater reductions in HbA1c (9.6 vs. 9.2), SBP (160 vs. 147), DBP (95 vs. 85), and LDL-C (148 vs. 128) compared to standard care group (p<0.05 for between-group differences) |  |  |  |  |  |
| **Ridgeway et al., 1999 [USA] [34]** | University Physicians Practice Group's ambulatory clinic in Kingsport, Tennessee | Randomised controlled trial | 56 | Service Delivery | Administration of education and health behaviour classes, delivered by nurse and dietitian, in a primary care setting | Control | - After 6 months, intervention group had reductions in mean FBG (from 215 to 180mg/dl, p=0.024), mean glycated hemoglobin (12.28% to 10.21%, p=0.034), mean LDL-C (133 to 113 mg/dl, p=0.313), and mean total cholesterol (59 to 221 mg/dl, p=0.0129) |  |  |  |  |  |
| **Russell et al., 2013 [Australia] [50]** | The catchment area of Inala Primary Care within Brisbane, Australia, which is a lower socio-economic suburb of outer urban Brisbane with a high ethnic and indigenous population | Trial | 328 | Service Delivery | Management of complex T2DM patients via a novel integrated primary/specialist model of community care which includes a multidisciplinary clinic, screening by a diabetes nurse educator, development of a patient-specific management plan by a clinical fellow, referrals to allied health professionals, ambulatory insulin stabilisation service, patient reviews of management plans at specific intervals | Control | - Mean HbA1c in intervention group decreased from 70.4 mmol/mol to 60.7 mmol/mol at 12 months (mean difference -9.0; 95% CI -12.2 to -6.4, p<0.05) - After stratification into quartiles based on baseline HbA1c, the intervention group had lower HbA1c after 6 months - % participants in intervention group achieving HbA1c target of ≤53 mmol/mol (7%) increased from 21 to 42% (P<0.001); no significant increase in usual care group |  |  |  |  |  |
| **Simpson et al., 2011 [Canada] [42]** | Five primary care clinics affiliated with the Edmonton South Side Primary Care Network in Edmonton, Alberta | Randomised controlled trial | 260 | Inputs (Human resource) | Addition of pharmacist to primary care team | Control | - Over 1 year, reduction in SBP for intervention patients (-7.4mmHg, 95%CI 4.6-10.2, p<0.001) but no significant reduction in control patients who received usual care by primary care team - Between-group difference in SBP at 1 year in favour of intervention (4.9mmHg, 95%CI 1.0-8.7, p=0.01) - OR 2.55 (95%CI 1.3 – 5.01, p=0.0065) for 10% decrease in SBP |  |  |  |  |  |
| **Smith et al., 2011 [Ireland] [46]** | 20 general practices in the east of the Republic of Ireland | Cluster Randomised controlled trial | 395 | Inputs (Human resource) | Standardised diabetes care system with 2-year peer-support intervention that contained four elements: the recruitment and training of peer supporters, nine group meetings led by peer supporters in participant’s own general practice, and a retention plan for peer supporters | Control | - At two-year follow-up, no significant differences in HbA1c, SBP, total cholesterol despite trend towards decreases in proportion of patients with poorly controlled risk factors at follow-up |  |  |  |  |  |
| **Taylor et al., 2003 [USA] [35]** | Community-based family clinics located in Aliceville and Gordo in Pickens County, Alabama (areas with severe poverty, low insurance coverage, poor health indicators), and affiliated with the University of Alabama School of Medicine - Tuscaloosa | Randomised controlled trial | 69 | Inputs (Human resource) | Pharmaceutical care initiative, including a medical record review, a medication history review, pharmacotherapeutic evaluation, and patient medication education and monitoring over a one-year period in community-based physician offices | Control | - At 12 months, intervention-group patients more likely than control patients to achieve blood pressure (BP) targets (intervention 91.7% vs. control 27.6%, p=0.001) - At 12 months, increase from baseline in the percentage of patients at BP (12.5% vs 91.7%, p<0.001), lipid (10.5% vs 77.8%, p<0.001) goals in intervention group - Control group received standard medical care. |  |  |  |  |  |
| **Yuan X, 2016 [China] [43]** | Hospital in Eastern China | Randomised controlled trial | 120 | Service Delivery | Case management of diabetic patients in outpatient settings, based on an evidence-based protocol focused on behaviour change | Control | - HbA1c reduced in CM group compared to control group at 6 months compared to baseline, with least mean of 0.43 (95% CI: 0.83, 0.03, p=0.034) - Statistically significant reductions did not persist at 12 and 24 months - % of participants with HbA1c ≤7.0% was higher over time in the CM group (45.5% at baseline, 54.5% at 6 months, 60.0% at 12 months, and 61.8% at 24 months) - At 24 months, % of participants with HbA1c 7.0% higher in CM group than in control group (61.8% vs. 41.5%, P = 0.035) |  |  |  |  |  |

MIXED METHODS

| **Study (Author, Year, Setting)** | **Context** | **Study Design** | **Sample Size** | **Health System framework domain** | **Health System Arrangement** | **Outcomes** | **Relevant findings (95% confidence interval given in Parenthesis)** | **Risk of Bias (Observational)** | | | | **Risk of Bias (Qualitative)** |
| --- | --- | --- | --- | --- | --- | --- | --- | --- | --- | --- | --- | --- |
|  |  |  |  |  |  |  |  | S | D | N | C |  |
| **Collinsworth et al., 2013 [USA] [113]** | Community health worker led diabetes self-management education program (DSME) for uninsured and underserved patients | Mixed methods | 497 | Inputs (Human resource) | Community health workers delivering a diabetes education curriculum to hispanic patients | control | - Improved mean A1C value from 8.7% at to 7.4% following participation (p<0.001) - Improved mean SBP (baseline 129.8mmHg vs. 1-year follow-up 127.3, p=0.03) |  |  |  |  | Medium (7 yes on COREQ) |
| **Loskutova et al., 2016 [USA] [115]** | T2DM patients managed in primary care practices part of a quality improvement project | Mixed methods | 179 | Service Delivery | Patient Navigator Model: Telephone-based non-professional patient navigation for patients, who were knowledgeable of the community resources, provided feedback to providers, patients and community programs and maintained a patient navigation tracking database using a web-based client relationship management system | Control | - Compared with baseline, reduction in HbA1c after the intervention (7.8 vs 7.2%, p=0.001) among subgroup of patients with an existing diagnosis of T2DM |  |  |  |  | Low (8 yes on COREQ) |
| **Katz et al., 2009 [South Africa] [114]** | Primary Health care nurses and patients incorporated into a program modelled on the chronic care model | Mixed methods | 257 | Service Delivery + Inputs (intellectual) | Role of Primary Healthcare nurses provided decision support, escalated scaling up of medication, and prompt access to specialist care | Control/Awareness | - Programme successful in supporting Primary Health Care Nurses (PHCN)s, detecting patients with advanced disease, and ensuring early referral to a specialist center - Programme improved early detection and referral of high risk, poorly controlled patients and had an impact on PHCNs’ knowledge - Disadvantages: poor follow up due to poor existing health systems and inability to integrate into existing chronic disease services - No clinical outcomes were reported |  |  |  |  | Low (8 yes on COREQ) |

QUALITATIVE

| **Study (Author, Year, Setting)** | **Context** | **Study Design** | **Sample Size** | **Health System framework domain** | **Health System Arrangement** | **Outcomes** | **Relevant findings (95% confidence interval given in Parenthesis)** | **Risk of Bias (COREQ)** |
| --- | --- | --- | --- | --- | --- | --- | --- | --- |
| **Alberti et al., 2007 [Tunisia] [108]** | People with Diabetes and Healthcare professionals in Primary care settings | Qualitative | 26 | Financing | Barriers to primary care management | Adherence | - Patients and health professionals quoted financial reasons as the cause of poor patient compliance (compliance in this study refers to adherence to diet, medications, blood tests and referrals) | Low (8 yes on COREQ) |
| **Bhojani U, 2013, [India] [109]** | T2DM sufferers in an urban slum  recruited through CHW | Qualitative | 16 | Financing | Constraints in managing T2DM | Adherence | - Financial constraints as major barrier to accessing chronic illness medication that should be taken for years or a lifetime | Low (8 yes on COREQ) |
| **Gazmararian et al., 2009 [USA] [110]** | Economically disadvantaged patients with diabetes | Qualitative | 35 | Financing | Barriers to achieving diabetes self-management | Adherence | - Cost not mentioned as a barrier to medication adherence | Low (8 yes on COREQ) |
| **Jeragh-Alhaddad FB, 2015 [Kuwait] [111]** | Patients with T2DM managed in GPs or hospitals managing diabetes with either oral medication or insulin or both | Qualitative | 20 | Financing | Barriers to medication taking | Adherence | - Unavailability of medications, difficulties accessing physicians and medications, inequalities in care provision and medication supply at different healthcare facilities, and lack of trust in the government healthcare system as barriers to medication adherence | Low (8 yes on COREQ) |
| **Lewis CP, 2014 [Bangladesh] [107]** | T2DM patients managed at various healthcare facilities | Qualitative | 23 patients, 8 HCP | Financing | Patient's perspectives of care | Awareness/Treatment | - Access to appropriate diagnosis and subsequent treatment was restricted by availability and costs of services | Medium (7 yes on COREQ) |
| **Mendenhall E, 2015 [South Africa] [112]** | Low-income black female T2DM patients | Qualitative | 27 | Financing | experiences of diabetes care | Adherence | - Structural barriers, e.g. overcrowded clinics and poor access to medicines, as impeding adherence to treatment | Low (8 yes on COREQ) |
