# Supplementary material for: Assessing the influence of health systems on Type 2 Diabetes Mellitus awareness, treatment, adherence, and control: A systematic review
Source: PLoS One. 2018 Mar 29;13(3):e0195086. doi: 10.1371/journal.pone.0195086 (PMC5875848; doi:10.1371/journal.pone.0195086)
Supplement: S2 Text — (DOCX) [file pone.0195086.s003.docx]

S2 Text. Medline search string

| *No.* | *String* | *No. of results* |
| --- | --- | --- |
| 1 | *type 2 diabetes/ | 85089 |
| 2 | *type 2 diabetes mellitus/ | 85089 |
| 3 | *adult onset diabetes/ | 85089 |
| 4 | ((high or increased or elevated) adj2 blood glucose).ab,ti. | 3131 |
| 5 | ((high or increased or elevated) adj2 blood sugar).ab,ti. | 403 |
| 6 | insulin resist*.ab,ti. | 5854 |
| 7 | hyperglycem*.ab,ti. | 35851 |
| 8 | (pre?diabet* or diabet*).ab,ti. | 452524 |
| 9 | 1 or 2 or 3 or 4 or 5 or 6 or 7 or 8 | 499861 |
| 10 | "equipment and supplies"/ | 22671 |
| 11 | *health facilities/ | 8076 |
| 12 | *glucose tolerance test/ | 5126 |
| 13 | *health services administration | 3156 |
| 14 | *health education/ | 32791 |
| 15 | *delivery of healthcare/ | 47905 |
| 16 | *primary healthcare/ | 41314 |
| 17 | *internet/ | 31756 |
| 18 | *health services accessibility/ | 29913 |
| 19 | *health planning/ | 11922 |
| 20 | *health priorities/ | 4747 |
| 21 | *"fees and charges"/ | 3831 |
| 22 | *insurance coverage/ | 5656 |
| 23 | *health services research/ | 13852 |
| 24 | *health status diparaties/ | 7019 |
| 25 | *Social support/ | 21679 |
| 26 | *financing, government/ | 8274 |
| 27 | 10 or 11 or 12 or 13 or 14 or 15 or 16 or 17 or 18 or 19 or 20 or 21 or 22 or 23 or 24 or 25 or 26 | 284195 |
| 28 | 9 and 27 | 8707 |
| 29 | (Diagnos* adj3 equipment).ab,ti. | 761 |
| 30 | (Health* adj2 (Facilities or facility)).ab,ti. | 15522 |
| 31 | Health record system*.ab,ti. | 454 |
| 32 | (Mobile unit* or glucometer or blood glucose meter or Consumable* or blood glucose measure* or blood sugar measure* or (Monitoring adj2 equipment)).ab,ti. | 3841 |
| 33 | ((Drug* or pharmaceutical* or medicine* or medicat*) adj3 (availability or affordability or appropriat* or cost*)).ab,ti.( | 20064 |
| 34 | ((Staff* or worker*) adj3 (train* or educat* or healthcare or model)).ab,ti. | 23041 |
| 35 | (Provide* adj1 educat*).ab,ti. | 3026 |
| 36 | (Polic* adj1 (maker* or planner* or manager*)).ab,ti. | 13155 |
| 37 | ((Inform* or empower*) adj2 (patient* or user* or consumer* or staff)).ab,ti. | 30383 |
| 38 | national guid*.ab,ti. | 5250 |
| 39 | (Inform* adj2 (self-care or health lifestyle* or diet* or physical activit*)).ab,ti. | 2294 |
| 40 | (Standard* adj2 (enforce* or implement* or monitor*)).ab,ti. | 3879 |
| 41 | "Rational drug use".ab,ti. | 234 |
| 42 | (Community adj3 (organisation* or mobili?ation)).ab,ti. | 1152 |
| 43 | ((Consumer or stakeholder) adj2 participation).ab,ti. | 424 |
| 44 | "Clinical governance".ab,ti. | 1045 |
| 45 | (Leadership or centrali* or decentrali*).ab,ti. | 42002 |
| 46 | ("policy authority" or (management adj2 approach*) or "quality assurance" or (setting adj2 practice) or "Chronic Care Model").ab,ti. | 31913 |
| 47 | (manag* adj2 (capacity or performance)).ab,ti. | 1594 |
| 48 | ((Legal or administrat*) adj2 framework).ab,ti. | 1142 |
| 49 | (Integrat* adj2 care).ab,ti. | 6021 |
| 50 | ((System or policy) adj2 design).ab,ti. | 2745 |
| 51 | (Taxation or "Targeted payment" or "targeted payments" or Commissioning or Capitation or Cost?sharing or Financing).ab,ti. | 15962 |
| 52 | (Fee adj2 service).ab,ti. | 3875 |
| 53 | (social adj2 (capital or support)).ab,ti. | 27353 |
| 54 | ((Social?health or Community?based health or Private) adj3 insurance).ab,ti. | 4664 |
| 55 | 29 or 30 or 31 or 32 or 33 or 34 or 35 or 36 or 37 or 38 or 39 or 40 or 41 or 42 or 43 or 44 or 45 or 46 or 47 or 48 or 49 or 50 or 51 or 52 or 53 or 54 | 247543 |
| 56 | 9 and 55 | 8359 |
| 57 | 28 or 56 | 15885 |
| 58 | ((Diagnos* or aware* or detect* or ident* or treat* or manag* or control* or adher* or screen*) adj3 (type 2 diabet* or hyperglycem* or insulin resist* or blood glucose or blood sugar)).ab,ti. | 27104 |
| 59 | ((medication or drug or treatment) adj3 (compliance or adherence)).ab,ti. | 23546 |
| 60 | exp medication adherence/ | 12475 |
| 61 | exp patient compliance/ | 64449 |
| 62 | 58 or 59 or 60 or 61 | 101418 |
| 63 | 57 and 62 | 2014 |
| 64 | (animals not (humans and animals)).sh. | 4288528 |
| 65 | (rat or rats or rodent* or mouse or mice or murine or dog or dogs or canine* or cat or cats or feline* or rabbit or rabbits or pig or pigs or porcine or swine or sheep or ovine* or guinea pig*).ti. | 1731263 |
| 66 | 64 or 65 | 4492754 |
| 67 | 63 not 66 | 1984 |
