# Supplementary material for: Assessing the influence of health systems on Type 2 Diabetes Mellitus awareness, treatment, adherence, and control: A systematic review
Source: PLoS One. 2018 Mar 29;13(3):e0195086. doi: 10.1371/journal.pone.0195086 (PMC5875848; doi:10.1371/journal.pone.0195086)
Supplement: S3 Text — (DOCX) [file pone.0195086.s004.docx]

S3 Text. Tool for assessing risk of bias for observational studies

| **Type of bias** | **Study design** | | | |
| --- | --- | --- | --- | --- |
|  | **Cross sectional** | **Case control** | **Cohort** | **Ecological** |
| Selection bias | Was the study population selected appropriate? | | | |
|  | Was the sample representative of its target population? | Were the controls randomly selected from the same population as the cases? | Was an appropriate control group used?  Was follow up sufficiently complete? (>80%) | Were the subjects representative of the group, place, or population of interest? |
| Differential misclassification | Did the assessment of the exposure or outcome differ according to the patient status? | Did the exposure assessment differ for cases and controls? | Did the outcome assessment differ for exposed and non-exposed? | Were the exposure and outcome variables measured and defined in the same or a similar way across the different groups studied? |
| Non-differential misclassification | Were valid methods used for measuring hypertension awareness, treatment or control and medication adherence? | | | |
| Confounding | Was any strategy undertaken to control for potential confounders?   1. At the design stage (restriction, matching) 2. At the analysis stage (stratification, multivariable analysis) | | | |

**Define each domain as low risk of bias, medium risk of bias or high risk of bias**
