# Supplementary material for: Assessing the influence of health systems on Type 2 Diabetes Mellitus awareness, treatment, adherence, and control: A systematic review
Source: PLoS One. 2018 Mar 29;13(3):e0195086. doi: 10.1371/journal.pone.0195086 (PMC5875848; doi:10.1371/journal.pone.0195086)
Supplement: S5 Text — (DOCX) [file pone.0195086.s006.docx]

S4 Text.Tool for quality appraisal of qualitative studies

| **Domain** | **Criteria for assessment** | **Yes or No** |
| --- | --- | --- |
| Background | Did the report provide an explanation and justification for the focus of the study and methods used?  Is there an explicit account of a supporting theoretical framework and/or a supporting literature review? |  |
| Aims and objectives of the study | Did the report explicitly and clearly state the aims of the study? |  |
| Context | Did the report adequately describe the specific circumstances under which the research was developed, carried out and completed? |  |
| Sampling | Did the report provide adequate details of the sampling strategy used, including methods for sampling and recruitment, and characteristics of subjects included in the study? |  |
| Description of data collection and analysis | Were both data collection and data analysis methods clearly described in the report? |  |
| Reliability of data analysis | Does the report provide evidence of attempts to demonstrate the reliability of data analysis? For example did more than one researcher check the coding or did the researchers present evidence that participant accounts have been faithfully represented? |  |
| Clarity about how conclusions were derived | Was sufficient original data - such as quotations, data tables or observations - included in the report to confirm the links made between data and interpretations or conclusions? |  |
| Reflexivity | Did the report reflect on the impact on the findings of the researchers own views and relationships with the participants? |  |
| Generalizability | Did the report outline the extent to which any findings can be generalised beyond the settings and participants of study and acknowledge the limits of generalizability? |  |
| Ethics | Was their evidence of consideration of ethical issues, such as confidentiality, in the report? |  |
